# Design of a cluster-randomized, hybrid type 1 effectiveness-implementation trial of a care navigation intervention to increase substance use disorder treatment engagement: study protocol

**DOI:** 10.1186/s13722-025-00605-7

**Published:** 2025-10-01

**Authors:** Theresa E. Matson, Mia A. Navarro, Abisola Idu, Jennifer F. Bobb, Briana M. Patrick, Rebecca Phillips, Tyler D. Barrett, Fernanda S. Rossi, Noa Krawczyk, Rachael Doud, Kristine Rogers, Chayna J. Davis, Ryan Caldeiro, Joseph E. Glass

**Affiliations:** 1 Kaiser Permanente Washington Health Research Institute, 1730 Minor Ave, Ste. 1300, Seattle, WA 98101 USA; 2 Center for Dissemination and Implementation at Stanford, Department of Psychiatry and Behavioral Sciences, Stanford University School of Medicine, Palo Alto, USA; 3 Center for Opioid Epidemiology and Policy, Department of Population Health, New York University Grossman School of Medicine, New York, USA; 4 Kaiser Permanente Washington Mental Health and Wellness Service, Seattle, USA; 5 Lighthouse Institute, Chestnut Health Systems, Eugene, USA

**Keywords:** Substance use disorder, Care navigation, Implementation, Protocol, Crossover trial

## Abstract

**Background:**

Practical and motivational barriers can deter people from engaging in substance use disorder (SUD) treatment, even those who seek treatment. Care navigation is a psychosocial intervention that seeks to facilitate patients’ timely access to care by identifying and intervening upon barriers*.* Few trials have tested the effectiveness of care navigation when embedding in real-world healthcare, and no trials have studied the process of implementing care navigation into clinical practice. This protocol describes a study that will evaluate whether care navigation can increase treatment engagement among patients seeking SUD treatment.

**Methods:**

The Addressing Barriers to Care for Substance Use Disorder (ABC-SUD) study is a hybrid type I cluster-randomized effectiveness-implementation trial. It is conducted in a mental health access center of an integrated healthcare system in Washington state. Within this center, licensed mental health clinicians assess patient needs and use shared decision-making to establish SUD treatment plans for patients (usual care). This study tests whether an added care navigation intervention can improve patient engagement in SUD treatment. Care navigation begins after a treatment plan is made and provides up to 7 weeks of support focused on enhancing patient motivation to initiate and engage in treatment, problem-solving barriers (e.g., transportation logistics), and accommodating patient preferences (e.g., preferred language of care, cultural preferences). This trial uses a two period, two sequence crossover design. Clinicians are randomized to offer care navigation to patients during the first or second study period (i.e., clinicians are assigned to an initial study condition and switch conditions halfway through the trial). Care navigation is implemented with several strategies: leadership engagement, clinical workflow specifications, electronic health record (EHR) tools, training, performance improvement, and electronic learning collaborative. The primary outcome—obtained from EHRs and insurance claims—is engagement in SUD treatment, defined as ≥3 SUD treatment visits within 48 days of a treatment plan. This study uses standardized measures of implementation climate and outcomes to examine mechanisms with which the intervention strategies exert their impact on implementation and effectiveness outcomes.

**Discussion:**

The ABC-SUD study will test whether care navigation improves SUD treatment engagement while concurrently generating information about its implementation in healthcare.

**Trial registration:**

This study was prospectively registered at www.clinicaltrials.gov (NCT06729957) on December 9, 2024.

**Supplementary Information:**

The online version contains supplementary material available at 10.1186/s13722-025-00605-7.

## Introduction

There is a critical addiction epidemic [1, 2]. In 2021, approximately 265 people a day in the United States died from drug overdose. Unhealthy alcohol and drug use costs the U.S. healthcare system over $38 billion annually [3].

Various effective interventions for substance use disorder (SUD) can be implemented in healthcare and community settings. However, only about 8–14% of people with past-year SUD receive any treatment [4, 5]. Some epidemiologic studies found that professionally delivered psychosocial SUD treatments and medications for SUD were less likely to be delivered to populations with access to fewer resources, such as people living in rural areas and people of minoritized racial and ethnic groups [6, 7]. Numerous studies have shown that people with SUD who self-identified a need for treatment reported not attending treatment due to practical and motivational barriers (e.g., time constraints, unaffordable treatment, shame around discussing SUD or requiring assistance with SUD treatment) [8, 9].

There has been a recent influx of SUD-focused psychosocial intervention studies that identified patients in a healthcare setting who need SUD treatment and provided patients assistance with initiating and engaging in SUD treatment. One successful family of interventions that has been validated in numerous patient populations, including patients with SUD, is called *care navigation* (also called *patient navigation*) [10]*.*

Care navigation is a psychosocial intervention approach that seeks to facilitate timely patient access to appropriate care by identifying and intervening upon a patient’s barriers to obtaining treatment [10]*.* SUD-focused care navigation interventions have sought to help patients identify and problem-solve logistical barriers to obtaining treatment. This may involve supportive follow-up calls and text messages, treatment center coordination assistance, and linkage to resources for basic needs (e.g., transportation assistance) while helping patients maintain motivation for treatment seeking [11, 12]. Evidence suggests that interventions that incorporate principles of care navigation for people with SUD are effective in initiating more patients to SUD treatment [12–14], prevent hospital readmissions [15], and are cost-saving [15].

While there is evidence that care navigation interventions increase SUD treatment initiation, real-world implementation and sustainability of such interventions is unclear. We are unaware of trials that have studied the process of implementing such interventions into routine clinical practice, nor are we aware of trials that have comprehensively evaluated the implementation context while testing the effectiveness of care navigation compared to usual care in an integrated healthcare system. In this study, we partnered with care delivery leaders in an integrated healthcare system to evaluate the implementation and effectiveness of care navigation for SUD within a mental health access center. Centralized treatment access centers, which exist internationally [16–20], perform the following key activities: field calls from patients seeking treatment, process treatment referrals, provide insurance authorizations, assist patients with issues of payment for care, provide contact information for treatment, and schedule assessment and treatment appointments. As a result, treatment access centers may have limited ability to address the social, motivational, and logistical needs and preferences of patients which fluctuate over time. Implementing care navigation, which follows patients in the community and helps them address barriers to initiation and engagement, is critical for the high volume of patients who require extra assistance to initiate and continue SUD treatment.

### The ABC-SUD study

The Addressing Barriers to Care for Substance Use Disorder (ABC-SUD) study is a hybrid type 1 cluster-randomized trial designed to evaluate the clinical effectiveness of a care navigation intervention while generating information about how to optimally implement care navigation in routine care. The intervention is embedded within a mental health access center in a large healthcare system. The limited evidence on the effectiveness of SUD care navigation in large integrated healthcare systems—combined with minimal guidance on its implementation—justifies a hybrid type 1 design that prioritizes effectiveness outcomes while rigorously measuring implementation processes such as the implementation context, progression through stages of implementation completion, and implementation costs [21]. This simultaneous evaluation allows implementers to more rapidly identify critical aspects of the protocol or implementation processes necessary for achieving clinical effectiveness in new settings [22].

The ABC-SUD study is one of three research projects within the Center for Dissemination and Implementation at Stanford (C-DIAS), a Research Center of Excellence focused on implementation science. Each project uses common implementation tracking tools and measures to promote transferable knowledge about deploying evidence-based addiction treatment in real-world settings.

#### Specific aims

The first aim is to evaluate whether care navigation increases engagement in SUD treatment among patients who contact a mental health treatment access center to seek care for SUD.

The second aim is to describe the implementation of care navigation, including the implementation process, patient and clinician perceptions about contextual factors and the intervention, and implementation outcomes.

## Methods/design

### Setting

The study is conducted in a mental health access center of Kaiser Permanente Washington (KPWA), a health insurance coverage and delivery system that serves a patient population (~700,000) with private, Medicaid, and Medicare insurance. Most members receive their primary care in one of 25 KPWA clinics located in communities across Washington state. Patient populations of the clinics vary in racial and ethnic diversity; most enrolled patients are white, yet across clinics, approximately 13-53% of patients are Black/African American, American Indian/Alaskan Native, Asian, Native Hawaiian/Pacific Islander, or Hispanic/Latino. KPWA also serves a large population that is covered by KPWA insurance but receives primary care elsewhere (i.e., from agencies/providers in a contracted network).

The mental health access center is a virtual triage center. KPWA patients who are seeking SUD treatment or mental health care may call the mental health access center directly (i.e., self-referral). Some patients receive outreach from the mental health access center, usually when referred by another provider in urgent cases. Once connected, master’s-level licensed mental health clinicians (‘care coordinators’) conduct mental health assessments over phone or video call, create a treatment plan with patients, appoint patients to internal providers within the healthcare system and/or refer patients to external agencies in the contracted network (see Usual Care below). Internally, patients may receive specialty SUD treatment from KPWA’s addiction recovery service, mental health treatment from licensed therapists in KPWA’s mental health and wellness department, or medications from KPWA primary care physicians and psychiatrists. When internal treatment is not available (e.g., no appointments), accessible (e.g., treatment is too far away), or appropriate (e.g., higher-acuity care is needed), patients are referred to external agencies in the community that accept KPWA insurance.

### Overview of trial design

**Fig. 1:**
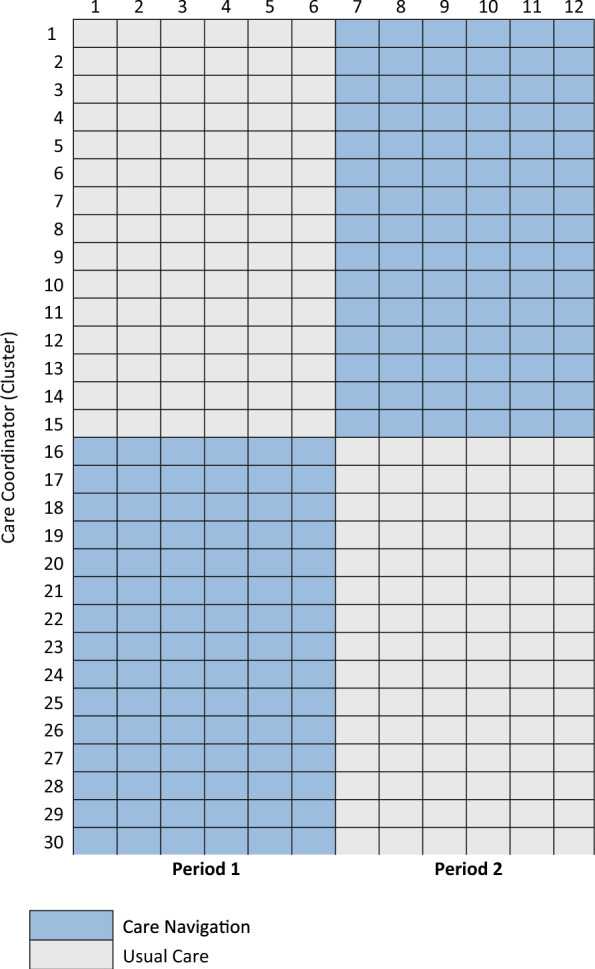
Two-period, two-sequence cluster-randomized crossover trial

The trial uses a two period, two sequence, cluster-randomized crossover trial design [23] with care coordinators as the clusters (Fig. 1). In this design, care coordinators receive both intervention and usual care conditions in a random order. Care coordinators are randomized to offer care navigation or offer usual care for the first period. Care coordinators switch conditions for the second period. The ability to offer care navigation is controlled by prompts embedded in the electronic health record (EHR) that will be turned on and off for each care coordinator by the health system depending on a care coordinator’s assignment. As such, carryover of the intervention when care coordinators switch conditions is not expected and there is no washout between periods [23]. Each period is anticipated to be six months, though it may be extended to seven months to meet patient accrual goals.

Cluster-randomization was chosen because care delivery leaders deemed patient-level randomization to be infeasible, as protocolized workflows and cognitive burden preclude a single care coordinator from offering care navigation to some patients and not others on a case-by-case basis. Additionally, the crossover trial design provides greater control for potential imbalance in cluster-level baseline covariates and maximizes statistical power by reducing statistical inefficiencies associated with parallel-group cluster-randomized trials [23]. The study will compare the primary outcome across study periods in which care coordinators are assigned to the intervention versus the usual care condition.

#### Clinician eligibility criteria

Care coordinators are eligible if they conduct video- or phone-based assessment and treatment planning visits, have been employed for at least one month, and have completed training relevant to their role in the health system (estimated *n*=30). All eligible care coordinators are invited via email by the study’s lead care navigator, and those that consent to participate are enrolled in the trial. To maximize the number of eligible care coordinators, those who are newly hired at the study site can be enrolled at any time from study start through approximately two months before patient enrollment is complete. Whether care coordinators experience both intervention and usual care conditions will depend on the timing of their eligibility and randomization date (see Additional file 1).

#### Patient eligibility criteria

Patients are eligible if they are an adult (≥18 years), have an assessment visit with an enrolled care coordinator, and receive an SUD treatment plan at that visit. This study uses secondary administrative data for analytic sample identification and outcome measurement (see *Study Sample* for additional analytic exclusions). Only patients participating in care navigation activities must consent (see *Experimental Intervention: Care Navigation*).

#### Ethical considerations

Ethical approvals were sought from the KPWA Institutional Review Board (IRB). The KPWA Institutional Review Board granted a waiver of consent and HIPAA authorization to identify patients and conduct trial outcome analyses. A waiver of written consent was granted to enroll patients in care navigation. This study followed the Standard Protocol Items: Recommendations for Intervention Trials (Additional file 2) and the CONSORT extension for cluster-randomized trials. Table 1 provides the schedule of enrollment, interventions, and assessments.

**Table 1:** Timing of the ABC-SUD Trial’s assessment schedules, following SPIRIT

TIMEPOINT	**STUDY PERIOD**				
	Enrollment	Allocation	Post-allocation^1^		Close-out
	*–t*_*1*_	0	*t*_*1*_	*t*_*2*_	*t*_*x*_
**ENROLLMENT**^**2**^**:**					
**Clinician eligibility determination**	X				
**Informed consent**	X				
**Allocation**		X			
**INTERVENTIONS:**					
***care navigation – usual care***			X	O	
***usual care – care navigation***			O	X	
**ASSESSMENTS:**					
***Qualitative interviews***	X	X			
***Covariates***^***3***^	X	X	X	X	
***FAA/ILS/ICS***^***4***^			X	X	
***IFASIS***^***5***^			X	X	
***SIC/COINS (costs)***			X	X	
***Implementation outcomes*** ***(reach, adoption, fidelity)***			X	X	
***Effectiveness outcomes***^***6***^ ***(treatment engagement, initiation)***			X	X	X
***Sustainment (maintenance)***					X

COINS=costs of implementing new strategies; FAA=feasibility acceptability and appropriateness measures; ICS=implementation climate scale; IFASIS=inventory of factors affecting successful implementation and sustainment; ILS=implementation leadership scale; SIC =stages of implementation completion; SPIRIT=Standard Protocol Items: Recommendations for Intervention Trials

^1^t1 and t2 are discrete 6-month time periods

^2^Enrollment rows reflect cluster-level (care coordinators) enrollment. This study uses an open cohort where all patients visiting care coordinators can be identified as eligible on ongoing basis

^3^Patient covariates are assessed in the year prior to a patient’s qualifying visit from automatic data sources

^4^FAA/ICS/ILS are collected twice, once in each post-allocation time period, using clinician-level surveys

^5^IFASIS collected twice, once in each post-allocation time period using structured focus groups

^6^Collected from automatic data sources

### Randomization procedures

Eligible care coordinators are randomized 1:1 to one of two allocation sequences (intervention then usual care, or usual care then intervention) as they become eligible using a computer-generated list of random numbers created by the study biostatisticians. We will employ a permuted block randomization using variable (random) block sizes of 2 and 4 so allocations will be approximately balanced over time. Given that the crossover design balances baseline clinician covariates across intervention conditions (provided clinicians remain in the study for the full follow-up period), we will not stratify randomization on baseline clinician-level covariates. Group assignments will be concealed in a password protected file by the biostatisticians until care coordinators become eligible. The study is not blinded, but all outcomes are extracted from EHR and healthcare claims data and cannot be manipulated by the study team.

### Data sources

Data for primary outcomes evaluation are drawn from multiple sources, including EHR data and health plan insurance claims. EHR and claims databases include information generated by patient visits inside and outside of the KPWA healthcare system, respectively. Data domains include demographic characteristics, diagnosis codes, procedure codes, medications, visit type and location, and provider type. EHR databases additionally include behavioral health assessment and treatment plan data. See *Implementation Evaluation* for a description of additional data sources. Data will be stored on encrypted, firewalled, and password-protected servers.

### Formative design and feasibility testing

#### Study redesign

The trial design and focus on care navigation were selected in partnership with health system leaders, including a study co-investigator who is the health system’s medical director of addictions care, and service line and departmental managers at the study site. The study design evolved between the time of grant submission, funding, and study launch due to the external environment and practice ecosystem factors. To integrate partner preferences into intervention and clinical workflow design, we conducted 20 qualitative interviews with patients who sought SUD treatment, and 8 qualitative interviews with care coordinators. Interview domains included background information to inform intervention design, feedback on study and intervention design, and implementation preparation. Rapid analysis [24] of these data informed changes to the clinical intervention, workflow, and implementation strategies.

#### Pilot study

To test feasibility of the study redesign, we conducted a parallel-group, cluster-randomized pilot feasibility trial in preparation for the randomized controlled trial (pilot study ClinicalTrials.gov identifier: NCT06317987). The patient eligibility periods for the pilot and trial period are distinct. Pilot goals were to confirm patient eligibility criteria, to confirm selection and measurement of the primary and secondary outcome using EHR data, to confirm the feasibility and acceptability of randomization procedures [25], to evaluate feasibility of care navigation protocol delivery by care coordinators, and to evaluate the feasibility of EHR templates to assist care coordinators when referring patients to care navigators. The pilot also helped identify study implementation strategy needs and statistical analysis plans for the trial. The pilot’s recruitment goal was to offer care navigation to patients until a total of 10 patients agreed to care navigation. We conducted post-intervention, user-centered design interviews with pilot-enrolled patients to obtain intervention design feedback to inform care navigation intervention changes. Details of the pilot are available in Additional file 3.

### Interventions

#### Usual care

Under usual care at the study site, patients calling the mental health access center seeking SUD treatment and in need of a psychosocial assessment will have an encounter with a care coordinator. Care coordinators craft a treatment plan for the patient within a 60-minute appointment. The treatment plan can include a referral to a specific SUD treatment center or a list of treatment centers that the patient should contact to obtain an appointment. Patients lack longitudinal contact with care coordinators. No further action will be taken in the usual care condition.

#### Experimental intervention: care navigation

**Fig. 2:**
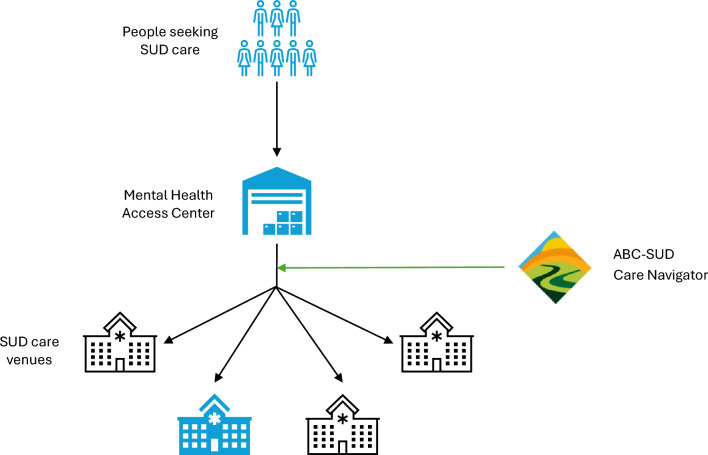
Care navigation in the study setting. Graphical depiction of the how patients flow through the study setting and where care navigation fits into this process. Blue shading depicts services/venues internal to Kaiser Permanente Washington. Abbreviations: ABC-SUD=Addressing Barriers to Care for Substance Use Disorder; SUD=substance use disorder

**Fig. 3:**
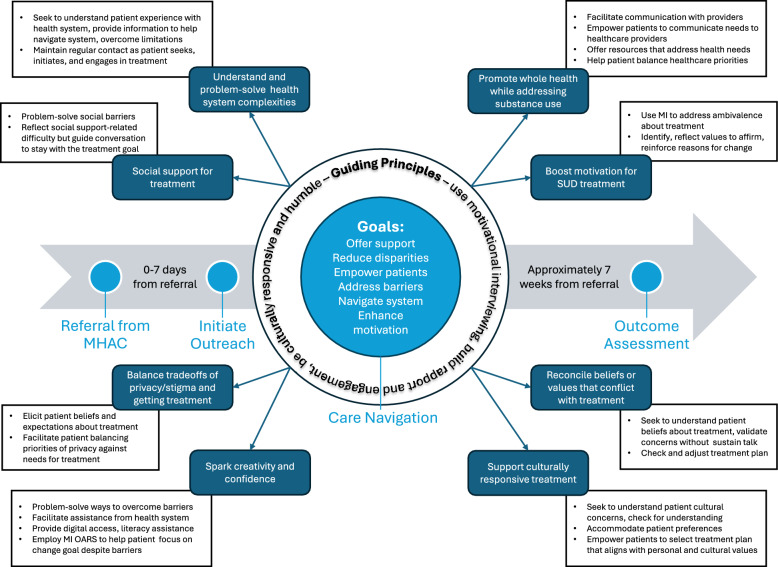
The ABC-SUD care navigation intervention. Care navigation goals (light blue) and principles guide every encounter between a care navigator and a patient. Core functions (dark blue) represent the common purpose of intervention activities that may vary across patients depending on their need and treatment plan. Intervention forms (white boxes) are the specific activities used to carry out core functions. The flow of care navigation activities is denoted by the horizontal arrow

After completing the assessment appointment as described above, care coordinators randomized to the intervention condition offer patients the option to speak with one of two ABC-SUD care navigators who can assist them while they engage in SUD treatment (Fig. 2). Care navigators are licensed mental health clinicians (RP and TDB) hired by the research study. If a patient agrees to receive further information about the care navigation service, care coordinators place a referral for care navigation through the EHR. The study’s lead care navigator receives the referral and conducts a brief chart review of the referred patient to confirm eligibility (i.e., patient is an adult, was referred by a trial-enrolled care coordinator and is seeking SUD treatment), then assigns the patient to one of the two care navigators’ caseloads. The assignment is based on care navigator availability, caseload balance, and patient preferences, if any. The care navigator then reaches out to patients via phone and/or secure message within seven days to explain the purpose of the study, obtain verbal consent (Additional file 4), and initiate navigation. The care navigator follows a standardized intervention manual. While the goals, guiding principles, and core functions of care navigation remain the same for all patients, the exact intervention forms are tailored to each patient’s needs and treatment plan (Fig. 3). The care navigator conducts outreach via phone and secure message as needed until the patient has initiated and engaged with treatment, outreach is exhausted, or the intervention period ends (seven weeks). We anticipate that each care navigator will be actively navigating ~10 patients at any given time over the course of the study.

To assist with fidelity monitoring and planful adaptations, we formalized the features of care navigation using the ‘core functions and forms’ framework (Additional file 5) [26]. *Core functions* describe the ultimate purpose of an intervention. *Forms* describe the specific activities used to carry out the intervention’s core functions (“intervention forms”). Core functions of care navigation were selected to address common needs and barriers of patients seeking SUD treatment [11, 13, 27–38]. Intervention forms were selected to reflect the core components of care navigation and common intervention procedures [10] and were matched to appropriate core functions. For example, one core function of care navigation is to address logistical barriers to care. Intervention forms include problem-solving ways to overcome these logistical barriers or offering resources (e.g., transportation resources, translation services, digital device loans) to help address barriers. Fully understanding the core functions of care navigation can help care navigators maintain intervention fidelity when tailoring interventions to the patient. While the ABC-SUD care navigation intervention has several pre-specified intervention forms (Additional file 5), other intervention forms could be applied to enact the core functions. This encourages a creative application of care navigators’ training and clinical instincts to deliver a patient-centered, fidelity-consistent care navigation intervention. As part of fidelity monitoring, care navigators track the activities of each care navigation encounter using a checklist [39] based on the core functions and forms framework [26] stored in a password-protected REDCap (Research Electronic Data Capture) database [40, 41]. Monthly, care navigators present cases to the PI as part of clinical supervision, describe fidelity ratings for the cases, discuss whether ratings were appropriate, and receive feedback on whether care navigation is being delivered to fidelity based on the case presentations.

Strategies to support cluster-level implementation of care navigation in the mental health access center are described below in *Implementation Strategies*.

### Randomized controlled trial evaluation

#### Study sample

The analytic sample contains all eligible patients who visit enrolled care coordinators (see *Patient Eligibility Criteria* above), regardless of whether they were offered and/or accepted care navigation. Patients that have a care coordinator encounter but are not enrolled in a KPWA health plan (includes commercial plans from employers, Medicare offered by KPWA, individual or family plans through Washington’s Health Benefit, or Medicaid via KPWA’s contract with Molina) will be excluded due to incomplete capture of covariate and outcome information.

#### Outcome measures

The primary effectiveness outcome is a binary measure of SUD treatment engagement, defined as completing ≥3 visits for SUD within 48 days of a mental health access center intake visit (Table 2). Treatment visits could occur within KPWA or the contracted care network. This outcome reflects the goal of care navigation to increase SUD care engagement and is consistent with the dose and timeframe of national quality metrics [42] used across health plans. Research suggests that this engagement metric predicts better outcomes [43]. To operationalize SUD treatment, we will use diagnosis codes for SUD when accompanied by relevant procedure codes for outpatient care or revenue codes for inpatient care obtained from the 2023 Quality Rating System HEDIS Value Set Directory [44] as well as national drug codes (for pharmacotherapy) from the 2023 Medication List Directory. All HEDIS codes were reviewed and refined by two researchers for applicability to SUD care in the KPWA health setting. Treatment will also include setting-specific encounters in KPWA’s internal addiction recovery services, regardless of procedure codes used. We anticipate adequate capture of SUD treatment for enrolled patients because billed services received outside of KPWA are captured using insurance claims.

**Table 2:** Primary, secondary, and other additional outcomes

Outcome	Definition (numerator/denominator)	Type
**Primary outcome**		
SUD treatment engagement^1,2^	Completed ≥3 SUD treatment visits within 48 days of index visit/all eligible patients	Binary
**Secondary outcome**		
SUD treatment initiation in 30 days^2,3^	Completed ≥1 SUD treatment visit within 30 days of index visit/all eligible patients	Binary
**Additional outcomes**		
SUD treatment initiation in 14 days	Completed ≥1 SUD treatment visit within 14 days of index visit/all eligible patients	Binary
Any SUD care in 14 days	Completed ≥1 SUD treatment visit and/or ≥1 visit treating SUD/all eligible patients sequelae within 14 days of index visit	Binary
Any SUD care in 30 days	Completed ≥1 SUD treatment visit and/or ≥1 visit treating SUD sequelae within 30 days of index visit/all eligible patients	Binary
SUD treatment initiation in 60 days	Completed ≥1 SUD treatment visit within 60 days of index visit/all eligible patients	Binary
Any SUD care in 60 days	Completed ≥1 SUD treatment visit and/or ≥1 visit treating SUD sequelae within 60 days of index visit/all eligible patients	Binary
SUD treatment initiation in 90 days	Completed ≥1 SUD treatment visit within 90 days of index visit/all eligible patients	Binary
Any SUD care in 90 days	Completed ≥1 SUD treatment visit and/or ≥1 visit treating SUD sequelae within 90 days of index visit/all eligible patients	Binary

^1^Primary effectiveness outcome is consistent with the Healthcare Effectiveness Data and Information Set timeframes for initiation + engagement

^2^Prospectively registered on clinicaltrials.gov

^3^Secondary effectiveness outcome reflects timeframes that may be more comparable with other studies

The secondary effectiveness outcome is a binary measure of SUD treatment initiation, defined as completing ≥1 visit for SUD within 30 days of a mental health access center intake (Table 2). This initiation outcome was modeled after several similar trials [45–47]. Additional pre-specified effectiveness outcomes (Table 2) will include measures that consider 1) different timeframes (i.e., ≥1 SUD visit within 14 [42], 60, 90 days of intake) and 2) a broader definition of SUD treatment sequelae (i.e., including detoxification, primary care or urgent care medical evaluations for withdrawal, crisis services, etc.).

Exploratory outcomes, time permitting, will include time until SUD treatment initiation, counts of SUD treatment days, and type of SUD treatment (i.e., behavioral health inpatient care, intensive outpatient programs, individual therapy or counseling, group therapy, pharmacotherapy, and evaluation or assessment visits).

See *Implementation Evaluation* for information about secondary implementation outcomes measurement.

#### Statistical analyses

Primary and secondary effectiveness outcome analyses will follow intent-to-treat principles, with care coordinators analyzed according to their assigned allocation sequence of intervention conditions regardless of the amount of care navigation delivered. We will use generalized linear mixed-effects models (GLMM). To account for clustering of patients within care coordinators, the model will include random intercepts for both cluster (care coordinator) and cluster period (periods 1 and 2) [23]. Including both random intercepts in the model allows for the possibility that individuals visiting the same care coordinator in the same period may be more strongly correlated than patients visiting the same care coordinator but in different periods [48]. The model will also include a main effect for period to allow for secular trends in the outcome over time [23]. Additional pre-specified binary effectiveness outcomes will be analyzed using the same approach. Time permitting, we will conduct a simulation study to explore the choice of statistical model, given that our trial setting with a small number of eligible patients per cluster and variable cluster sizes was not considered by prior statistical literature [23, 48, 49, 49–52]. If we find a model with improved performance in our setting, we will consider modifying our analytic approach accordingly; the final model will be specified in the Statistical Analysis Plan (Additional file 6) prior to obtaining outcome study data.

#### Exploratory outcome analyses

Time permitting, we will conduct exploratory analyses. This may include time-to-event analyses using Cox proportional hazard models [53] to understand differences in the length of time to initiate SUD treatment. Additionally, we may stratify analyses of outcomes by sex and gender, race and ethnicity, preferred healthcare language, insurance type, gender identity, age, urban/rural status, area-based measures (income, education, and unemployment levels), receipt of financial medical assistance, documented lack of housing, and SUD type, allowing us to provide data about effectiveness in subgroups, while recognizing our precision and power for subgroup analyses is limited [54].

#### Other considerations

We will examine crossover of patients between care coordinators and between usual care and intervention periods as outlined in the Statistical Analysis Plan (Additional file 6).

We expect patients who are enrolled in the KPWA health plan at the time of the index visit and who remain enrolled over the follow-up period will have complete capture of the primary outcome. For KPWA patients with Medicaid insurance, we will have EHR data on treatment received at KPWA but no claims data on treatment received externally. However, we will seek outcome data from Washington State to capture SUD treatment visits billed to Medicaid. If we cannot obtain Medicaid data, we will exclude Medicaid-enrolled patients from primary analyses. Patients not enrolled in KPWA health plans (defined above) are ineligible, because we cannot know when outcome data are missing or not.

Patients that disenroll from the health plan during the outcome follow-up period will provide incomplete outcome data. Should this occur, we will examine whether the proportion of patients who disenroll during the outcome follow-up period differs based on whether the patient visited during the intervention versus usual care period. If we observe that >10% patients disenroll during the outcome follow-up period, we will consider time-to-event analyses (see *Exploratory Outcome Analyses*), adjusting for baseline factors associated with disenrollment or the outcome.

It is also possible that some care coordinators randomized for the trial may leave the health system. We plan to examine whether care coordinators that drop out and those that continue in the study differ across baseline characteristics. We will adjust for baseline factors associated with drop out in the analysis.

#### Statistical power

Sample size and parameter estimates used for power calculations were estimated from preliminary data collected before the trial. With an assumed sample size of 30 care coordinators randomized (15 per allocation sequence), we estimated that we will have ≥80% power to detect an increase in the proportion of patients with SUD treatment engagement of ≥14.4 percentage points in the intervention arm compared to usual care, assuming a baseline outcome rate of 33% (estimated from preliminary data). This calculation assumed a mean sample size of 6 patients per cluster-period and that both the within-cluster-period and within-cluster-between-period correlations were 0 (estimated from preliminary data), with sensitivity scenarios considering alternate, non-null values for these correlations (Additional file 6).

Because not all eligible patients will participate in the care navigation intervention (e.g., missed offers by care coordinators and patients who decline the offer), any increases in SUD treatment engagement will be attenuated by those not participating in care navigation for whom we expect similar treatment engagement rates as seen in usual care (33%). If 50% of patients utilize care navigation, we need to observe a 28.8 percentage point increase in the proportion with treatment engagement (from 33% to 61.8%; Additional file 6). Although large, this effect is plausible based on a prior study of similar interventions [13].

### Safety and interim analyses

There is no data safety monitoring board and there are no formal interim analyses of safety or stopping guidelines given the low-risk nature of the intervention. All clinical care is provided as part of usual care, and care navigation seeks to direct patients to recommended services. The type and amount of care navigation will depend on patient preference. Patients may withdraw from the study at any time. The study team will report the following events to the IRB within 1 business day: 1) deaths that are both unanticipated and possibly related to the study, and 2) unauthorized use or disclosure of confidential information. Other reportable information will be reported to the IRB per institutional policies.

### Implementation approach and evaluation

#### Conceptual framework for enhancing implementation success

This proposed study integrates several conceptual frameworks. We used the Implementation Research Logic Model (IRLM) [55] to convey how each of these frameworks inform the design and implementation of care navigation (Additional file 7). Additionally, the IRLM provides a visual depiction of the relationship between the clinical intervention and its implementation. Briefly, the Health Equity Implementation Framework (HEIF; Additional file 7) [56] informs our understanding of contextual determinants and implementation mechanisms, the core functions and forms framework informs the care navigation protocol as previously described (see *Experimental Intervention*) [26], the Proctor et al. (2009) reporting guidelines inform how implementation strategies are specified (see Implementation Strategies) [57], and the RE-AIM framework (for Reach, Effectiveness, Adoption, Implementation, Maintenance) guides outcome selection (see *Implementation Outcomes*) [58]. The Exploration, Preparation, Implementation, Sustainment (EPIS) [59, 60] framework guides decisions and processes across the lifecycle of the study, as detailed in Additional file 7.

### Implementation strategies

We selected implementation strategies from prior literature [61] that improve adoption of care navigation by care coordinators and improve intervention delivery by care navigators. Care delivery-facing implementation strategies include leadership engagement, involving patients and care coordinators in intervention and workflow redesign, developing and implementing EHR tools for quality monitoring, clinician training, and performance improvement (e.g., partnering with clinicians and managers during the pilot to conduct small tests of change to improve the fit of care navigation intervention with the health system). These strategies focus on promoting implementation leadership behaviors that influence staff attitudes and commitment to successful implementation, co-designing EHR tools and clinical workflows that minimize burden on clinicians and patients, and providing feedback to clinicians on an going basis to improve performance [61]. For instance, we will deliver care navigation referral completion reports, enhanced by patient stories collected from qualitative interviews and the care navigator (“data and stories”), to managers and care coordinators to motivate care coordinators to utilize the care navigation program and engage in efforts to alleviate barriers to referring patients to care navigation [61]. Care navigator-facing implementation strategies include protocolizing procedures and workflows in intervention manuals, developing population dashboards for quality monitoring, self-monitored fidelity using a checklist, clinical supervision, and clinical training. These strategies focus on intervention fidelity to promote consistent delivery of high-quality care across patient encounters [39]. Specifications of each of these strategies are detailed in Additional files 8–9 according to Proctor et al. reporting guidelines [57].

#### Contextual implementation determinants

We will collect contextual implementation determinants data using surveys and structured focus groups with data stored in a password-protected REDCap database [40, 41]. A 42-item web-based survey will be sent out to care coordinators at up to three time points to collect demographic information and perceptions about the intervention and implementation context using psychometrically valid measures, including: 1) the Feasibility, Appropriateness, and Acceptability of Intervention Measures (FAA) [62]; 2) the Implementation Leadership Scale (ILS) [63]; and 3) the Implementation Climate Scale (ICS) [64]. These measures are described in Table 3.

**Table 3:** Implementation context measures

Measure	Administration	Description	Subscales/domains	Items	Responses	Target sample
The Feasibility, Appropriateness, and Acceptability of Intervention Measures [62]	Survey	Care coordinator perceptions about care navigation	Degree to which care navigation is: 1) acceptable (meets approval, appealing) 2) appropriate (fitting, suitable, applicable) 3) feasible (implementable, seems possible, doable, easy to use)	12	0 – complete disagree 1 – disagree 2 – neither agree nor disagree 3 – agree 4 – completely agree	All care coordinators
The Implementation Leadership Scale [63]	Survey	Care coordinator perception of leaders’ behavior in support of evidence-based practice implementation	Degree to which leadership is: 1) proactive (anticipates and addresses implementation challenges) 2) knowledgeable (has deep knowledge of EBP and implementation issues) 3) supportive (supports clinicians’ adoption and use of EBP) 4) perseverant (consistent, unwavering and responsive to EBP implementation issues)	12	0 – not at all 1 – slight extent 2 – moderate extent 3 – great extent 4 – very great extent	All care coordinators
Implementation Climate Scale [64]	Survey	Care coordinator perceptions of the importance of evidence-based practice (EBP) implementation within the organization	Degree to which organization climate has: 1) focus on EBP 2) educational support for EBP 3) recognition for EBP 4) rewards for EBP 5) selection for EBP 6) openness for EBP	18	0 – not at all 1 – slight extent 2 – moderate extent 4 – great extent 4 – very great extent	All care coordinators
Inventory of Factors Affecting Successful Implementation and Sustainment	Focus group	Care coordinator consensus on constructs within the health care system, and specifically the mental health access center department, that could influence efforts to implement care navigation	Focus group domains included: 1) factors outside the organization 2) factors within the organization 3) factors about care navigation 4) factors about the persons receiving care navigation	27	Status rating: 1 – nonexistent 2 – between 1 & 3 3 – mixed 4 – between 3 & 5 5 – strong Importance rating: 0 – not at all 1 – somewhat 2 - very	Purposive sample of 3-7 care coordinators
Qualitative interviews	1:1 Interviews	Patient and care coordinator feedback on barriers and facilitators to SUD treatment, the proposed care navigation intervention and its implementation	Interview domains included: 1) background information on multilevel barriers and facilitators to inform intervention design 2) feedback on study and intervention 3) implementation preparation	~9	Open-ended	Purposive sample of up to 30 patients and 30 care coordinators

We will conduct structured focus groups using the Inventory of Factors Affecting Successful Implementation and Sustainment (IFASIS) [65, 66] in small group settings of 3-7 care coordinators up to two times during the study period. The IFASIS is a comprehensive assessment tool that examines contextual factors influencing implementation efforts across four key domains: factors outside your organization, factors within your organization, factors about the intervention, and factors about the person receiving the intervention. These domains are further divided into 13 subdomains comprising 27 items [66]. For each item, participants rate two aspects: (1) valence—scored on a scale from 1 to 5, where 1-2 indicates a barrier, 3 indicates a neutral factor, and 4-5 indicates a facilitator; and (2) importance—rated on a scale from 1 to 3, where 1 is not important, 2 is somewhat important, and 3 is very important [66]. The goal is to foster discussion around each item and to reach a consensus among the group. This process yields both quantitative ratings and qualitative data about the factors affecting implementation, providing a nuanced understanding of the organizational context and potential challenges or facilitators for the implementation effort [65].

We will analyze qualitative interviews with patients and care coordinators conducted during the study redesign (see *Study Redesign*) to understand contextual determinants and implementation mechanisms.

#### Implementation process

We will use the Stages of Implementation Completion (SIC), a psychometrically valid and reliable tool used to define and measure implementation activities across implementation phases [67]. It enables diagnostics tools that compute normed scores based on the proportion of implementation activities completed and the amount of time spent completing activities. To track implementation strategy fidelity, key implementation strategy activities will be identified a priori, programmed into the SIC platform, and shared with health system leaders.

#### Implementation outcomes

Implementation outcomes are conceptualized via the RE-AIM framework (Table 4) [58]. These will include the proportion of all eligible patients in the intervention periods who agree to care navigation and have ≥1 care navigation visit (reach of care navigation), the proportion of all eligible care coordinators who enroll in the study (adoption intent by care coordinators), the proportion of enrolled care coordinators who offer care navigation to ≥2 eligible patients in the intervention periods (adoption of the intervention by enrolled care coordinators), and the proportion of all eligible patients in the intervention periods who agree to a care coordinator’s offer to be contacted by a care navigator (adoption intent by patients). Additional pre-specified implementation outcomes are outlined in Table 4.

**Table 4:** Implementation outcomes^1^

Outcome	Definition (numerator/denominator)	Type
Reach	Agreed to care navigation and had ≥1 care navigation visit/all eligible patients during the intervention periods	Binary
Adoption intent by care coordinators	Agreed to offer care navigation/all care coordinators invited to participate during the study period	Binary
Adoption by participating care coordinators	Offered care navigation to ≥2 eligible patients during the intervention periods/all care coordinators who agreed to participate	Binary
Adoption intent by patient	Agreed to the care coordinator offer to be contacted by a care navigator/all eligible patients during the intervention periods	Binary
Implementation, competency^2^	Received offer of care navigation/all eligible patients during the intervention periods	Binary
Implementation, intervention fidelity	Contacted by a care navigator within 7 days/all eligible and referred patients during the intervention periods	Binary
Implementation, intervention fidelity^2^	Needs that were met/all expressed needs among patients participating in care navigation intervention	Binary
Implementation, implementation fidelity	Number of months in which fidelity reviews occurred/all care navigation intervention months	Binary
Implementation, implementation fidelity	Number of months in which peer supervision occurred/all care navigation intervention months	Binary
Implementation, costs	Costs of implementing care navigation during the study	Continuous
Maintenance	Health system intent to adopt care navigation at the end of the study period (yes/no)	Binary

^1^Implementation outcomes were determined by RE-AIM, the Stages of Implementation Completion (SIC) and Costs of Implementing New Strategies (COINS)

^2^We will report whether and when competence/implementation fidelity reached ≥40%, ≥60%, ≥80% (binary measure for each threshold) to reflect growing competency/implementation fidelity over the course of the study

#### Implementation analysis

We will calculate descriptive statistics for implementation measures to describe the implementation climate and examine their associations with implementation outcomes.

#### Economic analysis

Implementation costs, measured from the health system perspective, are defined as the monetary value of time and resources needed to scale out care navigation to patients with SUD. We will collect data on costs separately for the system- and clinic-level strategies. This will provide scientists and healthcare decision-makers with information on the burden associated with enacting these strategies. We will apply the Cost of Implementing New Strategies (COINS) [68], which employs an implementation-phase based approach to assign units of cost to implementation activities (e.g., hourly wages from labor of KPWA employees, material costs). Implementation activities will be mapped onto SIC implementation stages and the SIC web platform will allow tracking of human resources (i.e., effort) and direct expenditures. The COINS and SIC tools are integrated to streamline the documentation of activities and costs in a single interface. A research team member will prospectively collect and enter data on time spent by care delivery leaders, implementers on the research team, programmers who create performance reports, and other personnel involved at the implementation sites.

### Trial status

**Fig. 4:**
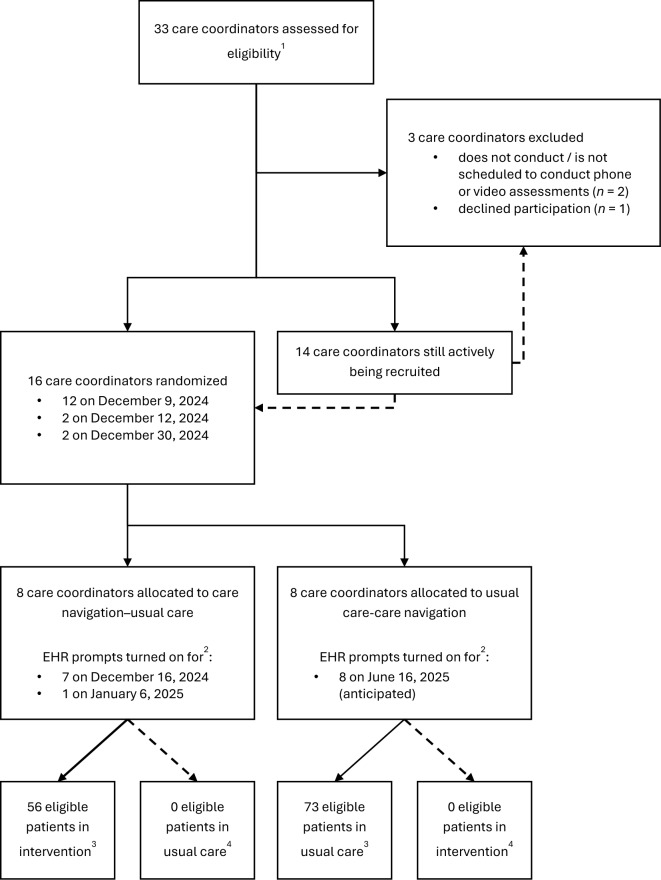
Current state of recruitment for ongoing study. CONSORT diagram of care coordinator enrollment and allocation to study conditions at present. CONSORT=Consolidated Standards of Reporting Trials; EHR=electronic health record ^1^The number of care coordinators assessed for eligibility may increase if new clinicians are hired into the care coordinator role during the study period ^2^The anticipated date that EHR prompts are turned off (for crossover and study end) is expected to be 6 months from the date the first patient was eligible ^3^Numbers reflect recruitment at the time of submission of this article; recruitment is ongoing ^4^Because care coordinators have not switched study conditions, there is no current accrual of eligible patients in the second study condition

The trial commenced on December 17, 2024. Care coordinator recruitment is ongoing (Fig. 4), and we will randomize additional care coordinators upon their enrollment in the trial. No outcome data have been analyzed yet. This manuscript serves as Version 1.0 of the study protocol, dated March 20, 2025. Protocol modifications will be updated in clinicaltrials.gov and changes to the Statistical Analysis Plan (Additional File 6) will be submitted with the final report.

## Discussion

While care navigation holds promise for many chronic conditions [69], the evidence for improving patient access and linkage to SUD treatment is limited to certain contexts and little information exists about how to implement and sustain care navigation in real-world health systems. This hybrid effectiveness-implementation trial will simultaneously test the effectiveness of care navigation for SUD in real-world clinical settings while also generating evidence that will inform the implementation and scalability in health systems that assess and refer patients to treatment. Moreover, care navigation was designed to be patient-centered, with interventions tailored to the needs and preferences of each patient. As such, care navigation may improve SUD treatment initiation and engagement for those who experience barriers due to lack of resources or systemic inequities [10].

There is broad acknowledgement in the field that effectiveness trials should seek to gather information about implementation early. This study serves as a model for gathering information early by engaging clinical partners, iteratively adapting the approach based on feedback and data, and embedding standardized implementation measures (i.e., FAA/ILS/ICS, IFASIS, SIC and COINS) to understand adaptability to other contexts. While this study is not testing the effectiveness of implementation strategies, we have designed our implementation strategies in a participatory way to improve the fielding of trial processes, the intervention itself, and how well the intervention interfaces with real-world care. This study applies and extends the concept of Design for Dissemination (D4D) [70]. This involves: 1) partner (end-user) engagement and co-design; 2) identifying contextual determinants of intervention use and adoption from patients and clinicians; 3) tracking the strategies being used, and their adaptations, to get people to use and take part in the intervention project; and 4) continuously measuring outcomes reach and adoption. To the extent that intervention developers employ these approaches, the chances for innovations, if proven effective, are more likely to be successfully translated into practice.

Finally, we note that given changes in the health system, this trial went through extensive redesign to factor in the needs and preferences of the health system, clinicians, and patients. While this process ostensibly takes time, we believe it was necessary to lay the foundation for new partnerships, develop the intervention with clinical partners, and ensure strong systems for data collection were in place.

### Strengths and limitations

This study has several strengths and limitations. Relying on administrative data to define analytic samples and assess outcomes allows us to calculate realistic measures of implementation success, such as the proportion of patients interested in receiving care navigation, while alleviating patient burden for participation in research. However, it raises several limitations. For instance, receipt of SUD treatment will not capture treatment that is not documented in EHRs or care that is not covered by insurance (e.g., Alcoholics Anonymous). We may not capture treatment for patients who disenroll from the health plan during the follow-up period but have planned alternative analyses if this is a common occurrence (see *Other Considerations*). This study uses a fidelity tool to track the functions and intervention forms of each care navigation encounter in a way that allows for flexible adaptation to individual patient needs. However, this study does not examine fidelity using audiotaped sessions, which is considered a gold-standard. While the study generates data on implementation costs, cost-effectiveness analyses are outside the scope of the study.

Additional multi-site studies are needed with larger cluster sizes and more diverse patient populations to understand the generalizability of care navigation and the implementation strategies needed to integrate them into systems. At least two factors should be considered when assessing the generalizability of this study. First, the health system in this study assesses and refers a large volume of patients for SUD treatment. Some treatment is provided by the health system itself, but most care is provided via a network of contracted care providers that provide specialty addiction services. Findings may generalize less well to systems that provide comprehensive addiction treatment services internally, such as the Veterans Health Administration, because fewer barriers to care may exist due to a lack of linkage across organizations. Second, all patients in this study have expressed interest in seeking SUD treatment. While motivation may vacillate even among treatment-seeking patients, and the care navigation approach incorporates motivational interviewing to maintain motivation for treatment, the intervention does not seek to motivate patients who are non-treatment-seeking. Findings may best generalize to settings that provide patients with information about treatment agencies following a psychosocial assessment, but do not currently have the capacity to follow the patient longitudinally to ensure that they have the support needed to initiate and engage in treatment.

## Conclusion

This study seeks to advance implementation science by understanding how a care navigation intervention and its implementation strategies enact their effects on SUD treatment engagement. As a foundational step, this study tests whether an intervention that addresses the many practical and logistical barriers to SUD treatment is effective while generating information about its implementation and sustainability in real-world settings. The use of standard implementation measures to rigorously document implementation climate will inform the development of scalable models that can be tailored to different healthcare systems and settings. This study has the potential to transform how healthcare systems deliver patient-centered support, not only for SUD treatment but also for other chronic conditions, ultimately improving care access, equity, and long-term health outcomes.

## Supplementary Information

Supplementary material 1: Time periods for mental health care coordinator (CC) and patient eligibility in the ABC-SUD trial.

Supplementary material 2: Standard Protocol Items: Recommendations for Intervention Trials (SPIRIT).

Supplementary material 3: Details of the ABC-SUD pilot.

Supplementary material 4: Study information sheet used for verbal consent of patients for care navigation.

Supplementary material 5: Map of patient needs/barriers expressed by people seeking substance use disorder treatment to the core functions of care navigation and pre-specified intervention forms.

Supplementary material 6: Statistical Analysis Plan.

Supplementary material 7: Conceptual Framework for Enhancing Implementation Success.

Supplementary material 8: Specification of care-delivery facing implementation strategies according to Proctor et al. (2011) reporting guidelines.

Supplementary material 9: Specification of interventionist-delivery facing implementation strategies according to Proctor et al. (2011) reporting guidelines.

## Data Availability

No datasets were generated or analysed during the current study.

## Abbreviations

ABC-SUD: Addressing Barriers to Care for Substance Use Disorder
C-DIAS: Center for Dissemination and Implementation at Stanford
COINS: Costs of Implementing New Strategies
CONSORT: Consolidated Standards of Reporting Trials
D4D: Design for dissemination
EBP: Evidence-based practice
EHR: Electronic health record
FAA: Feasibility, Acceptability, And Appropriateness
ICS: Implementation Climate Scale
IFASIS: Inventory of Factors Affecting Successful Implementation and Sustainment
ILS: Implementation Leadership Scale
IRB: Institutional Review Board
KPWA: Kaiser Permanente Washington
NIDA: National Institute on Drug Abuse
NIH: National Institutes of Health
PI: Principal investigator
REDCap: Research Electronic Data capture
SIC: Stages of Implementation Completion
SUD: Substance use disorder

## References

[1] Garnett MF, Miniño AM. Drug Overdose Deaths in the United States 2003-2023. [cited 2025 Feb 25]; Available from: https://stacks.cdc.gov/view/cdc/170565

[2] CDC. Alcohol Use. 2024 [cited 2025 Feb 25]. Facts About U.S. Deaths from Excessive Alcohol Use. Available from: https://www.cdc.gov/alcohol/facts-stats/index.html

[3] National Institute on Drug Abuse. Costs of Substance Abuse. 2020.

[4] Grant BF, Goldstein RB, Saha TD, Chou SP, Jung J, Zhang H, et al. Epidemiology of DSM-5 alcohol use disorder: results from the national epidemiologic survey on alcohol and related conditions III. JAMA Psychiatr. 2015;72(8):757–66. 10.1001/jamapsychiatry.2015.0584 PMC5240584 26039070

[5] Grant BF, Saha TD, Ruan WJ, Goldstein RB, Chou SP, Jung J, et al. Epidemiology of DSM-5 drug use disorder: results from the national epidemiologic survey on alcohol and related conditions-III. JAMA Psychiatr. 2016;73(1):39–47. 10.1001/jamapsychiatry.2015.2132 PMC5062605 26580136

[6] Chen JA, Glass JE, Bensley KMK, Goldberg SB, Lehavot K, Williams EC. Racial/ethnic and gender differences in receipt of brief intervention among patients with unhealthy alcohol use in the U.S. veterans health administration. J Subst Abuse Treat. 2020;119:108078. 32736926 10.1016/j.jsat.2020.108078 PMC7641963

[7] Lapham G, Boudreau DM, Johnson EA, Bobb JF, Matthews AG, McCormack J, et al. Prevalence and treatment of opioid use disorders among primary care patients in six health systems. Drug Alcohol Depend. 2020;207:107732. 31835068 10.1016/j.drugalcdep.2019.107732 PMC7158756

[8] Perron BE, Mowbray OP, Glass JE, Delva J, Vaughn MG, Howard MO. Differences in service utilization and barriers among Blacks, Hispanics, and Whites with drug use disorders. Subst Abuse Treat Prev Policy. 2009;4:3. 19284669 10.1186/1747-597X-4-3 PMC2660316

[9] Cohen E, Feinn R, Arias A, Kranzler HR. Alcohol treatment utilization: findings from the National Epidemiologic Survey on Alcohol and Related Conditions. Drug Alcohol Depend. 2007;86(2–3):214–21. 16919401 10.1016/j.drugalcdep.2006.06.008

[10] Freeman HP. The origin, evolution, and principles of patient navigation. Cancer Epidemiology, Biomarkers Prevention. 2012;21(10):1614–7. 10.1158/1055-9965.EPI-12-0982 23045534

[11] Krawczyk N, Rivera BD, Chang JE, Grivel M, Chen YH, Nagappala S, et al. Strategies to Support Substance Use Disorder Care Transitions From Acute-Care to Community-Based Settings: A Scoping Review and Typology [Internet]. Addiction Med. 2023. 10.1101/2023.04.24.23289042. 10.1186/s13722-023-00422-w PMC10621088 37919755

[12] James H, Morgan J, Ti L, Nolan S. Transitions in care between hospital and community settings for individuals with a substance use disorder: a systematic review. Drug Alcohol Depend. 2023;243(1):109763. 36634575 10.1016/j.drugalcdep.2023.109763

[13] Scott CK, Dennis ML, Grella CE, Watson DP, Davis JP, Hart MK. Using recovery management checkups for primary care to improve linkage to alcohol and other drug use treatment: a randomized controlled trial three month findings. Addiction. 2023;118(3):520–32. 36208061 10.1111/add.16064 PMC10015976

[14] Gryczynski J, Schwartz RP, Salkever DS, Mitchell SG, Jaffe JH. Patterns in admission delays to outpatient methadone treatment in the United States. J Subst Abuse Treat. 2011;41(4):431–9. 21821378 10.1016/j.jsat.2011.06.005 PMC3205308

[15] Orme S, Zarkin GA, Dunlap LJ, Nordeck CD, Schwartz RP, Mitchell SG, et al. Cost and cost savings of navigation services to avoid rehospitalization for a comorbid substance use disorder population. Med Care. 2022;60(8):631–5. 35687900 10.1097/MLR.0000000000001743 PMC9382857

[16] Di Renna T, Burke E, Bhatia A, Clarke H, Flamer D, Flannery J, et al. Improving access to chronic pain care with central referral and triage: the 6-year findings from a single-entry model. Can J Pain. 2024;8(1):2297561. 38562673 10.1080/24740527.2023.2297561 PMC10984115

[17] Wang J, Yang L, Tibbo P, Simon P, Bullerwell M. The health and psychosocial profiles of adults who sought mental health and addiction specialty services through a centralized intake process in Nova Scotia in 2020 and 2021. Can J Psychiatry. 2023;68(8):613–22. 36855805 10.1177/07067437231159768 PMC10411361

[18] Isaacs A, Bonsey A, Couch D. Centralized intake models and recommendations for their use in non-acute mental health services: a scoping review. Int J Environ Res Public Health. 2023;20(9):5747. 37174264 10.3390/ijerph20095747 PMC10177908

[19] Ala-Nikkola T, Pirkola S, Kaila M, Joffe G, Kontio R, Oranta O, et al. Identifying local and centralized mental health services-the development of a new categorizing variable. Int J Environ Res Public Health. 2018. 10.3390/ijerph15061131. 29857540 10.3390/ijerph15061131 PMC6025394

[20] Seattle Childrens. Mental Health Referral Service for Children and Teens in Washington [Internet]. [cited 2025 Mar 20]. Available from: https://www.seattlechildrens.org/clinics/washington-mental-health-referral-service/

[21] Curran GM, Bauer M, Mittman B, Pyne JM, Stetler C. Effectiveness-implementation hybrid designs: combining elements of clinical effectiveness and implementation research to enhance public health impact. Med Care. 2012;50(3):217–26. 22310560 10.1097/MLR.0b013e3182408812 PMC3731143

[22] Landes SJ, McBain SA, Curran GM. An introduction to effectiveness-implementation hybrid designs. Psychiatry Res. 2019;280:112513. 31434011 10.1016/j.psychres.2019.112513 PMC6779135

[23] Hemming K, Taljaard M, Weijer C, Forbes AB. Use of multiple period, cluster randomised, crossover trial designs for comparative effectiveness research. BMJ. 2020;4:m3800. 10.1136/bmj.m3800 33148538

[24] Hsu C, Mogk J, Hansell L, Glass J, Allen C. Rapid group analysis process (Rap-GAP): a novel approach to expedite qualitative health research data analysis. Int J Qual Meth. 2024. 10.1177/16094069241256275. 10.1177/16094069241256275 PMC12753008 41477558

[25] Margolis KL, Crain AL, Bergdall AR, Beran M, Anderson JP, Solberg LI, et al. Design of a pragmatic cluster-randomized trial comparing telehealth care and best practice clinic-based care for uncontrolled high blood pressure. Contemp Clin Trials. 2020;92:105939. 31981712 10.1016/j.cct.2020.105939

[26] Perez Jolles M, Lengnick-Hall R, Mittman BS. Core functions and forms of complex health interventions: a patient-centered medical home illustration. J Gen Intern Med. 2019;34(6):1032–8. 30623387 10.1007/s11606-018-4818-7 PMC6544719

[27] Frost MC, Matson TE, Richards JE, Lee AK, Achtmeyer CE, Bradley KA, et al. Barriers and facilitators to changing drinking and receiving alcohol-related care: interviews with veterans health administration primary care patients who indicated interest but did not enroll in an alcohol care management intervention trial. Subst Abus. 2022;43(1):1197–206. 35657656 10.1080/08897077.2022.2074602 PMC9555295

[28] Grigg J, Manning V, Cheetham A, Youssef G, Hall K, Baker AL, et al. A latent class analysis of perceived barriers to help-seeking among people with alcohol use problems presenting for telephone-delivered treatment. Alcohol Alcohol. 2022;58(1):68–75. 10.1093/alcalc/agac063 PMC9830485 36448844

[29] Browne T, Priester MA, Clone S, Iachini A, DeHart D, Hock R. Barriers and facilitators to substance use treatment in the rural South: a qualitative study. J Rural Health. 2016;32(1):92–101. 26184098 10.1111/jrh.12129

[30] Ross LE, Vigod S, Wishart J, Waese M, Spence JD, Oliver J, et al. Barriers and facilitators to primary care for people with mental health and/or substance use issues: a qualitative study. BMC Fam Pract. 2015;16(1):135. 26463083 10.1186/s12875-015-0353-3 PMC4604001

[31] Yang Y, Perkins DR, Stearns AE. Barriers and facilitators to treatment engagement among clients in inpatient substance abuse treatment. Qual Health Res. 2018;28(9):1474–85. 29683040 10.1177/1049732318771005

[32] Barnett ER, Knight E, Herman RJ, Amarakaran K, Jankowski MK. Difficult binds: a systematic review of facilitators and barriers to treatment among mothers with substance use disorders. J Subst Abuse Treat. 2021;126:108341. 34116826 10.1016/j.jsat.2021.108341

[33] Masson CL, Shopshire MS, Sen S, Hoffman KA, Hengl NS, Bartolome J, et al. Possible barriers to enrollment in substance abuse treatment among a diverse sample of Asian Americans and Pacific Islanders: opinions of treatment clients. J Subst Abuse Treat. 2013;44(3):309–15. 22985677 10.1016/j.jsat.2012.08.005 PMC3545039

[34] Manuel JI, Yuan Y, Herman D, Svikis D, Nichols O, Palmer E, et al. Barriers and facilitators to successful transition from long-term residential substance abuse treatment. J Subst Abuse Treat. 2017;74:16–22. 28132695 10.1016/j.jsat.2016.12.001 PMC5310811

[35] Timko C, Schultz NR, Britt J, Cucciare MA. Transitioning from detoxification to substance use disorder treatment: facilitators and barriers. J Subst Abuse Treat. 2016;70:64–72. 27692190 10.1016/j.jsat.2016.07.010 PMC6448765

[36] Farhoudian A, Razaghi E, Hooshyari Z, Noroozi A, Pilevari A, Mokri A, et al. Barriers and Facilitators to Substance Use Disorder Treatment: An Overview of Systematic Reviews. Subst Abuse. 2022;29(16):11782218221118462. 10.1177/11782218221118462 PMC9434658 36062252

[37] Trafton JA, Oliva EM, Horst DA, Minkel JD, Humphreys K. Treatment needs associated with pain in substance use disorder patients: implications for concurrent treatment. Drug Alcohol Depend. 2004;73(1):23–31. 14687956 10.1016/j.drugalcdep.2003.08.007

[38] Gryczynski J, Nordeck CD, Welsh C, Mitchell SG, O’Grady KE, Schwartz RP. Preventing hospital readmission for patients with comorbid substance use disorder: a randomized trial. Ann Intern Med. 2021;174(7):899–909. 33819055 10.7326/M20-5475 PMC13412153

[39] Akiba CF, Powell BJ, Pence BW, Nguyen MXB, Golin C, Go V. The case for prioritizing implementation strategy fidelity measurement: benefits and challenges. Transl Behav Med. 2021;12(2):335–42. 10.1093/tbm/ibab138 PMC8849000 34791480

[40] Harris PA, Taylor R, Thielke R, Payne J, Gonzalez N, Conde JG. Research electronic data capture (REDCap)—a metadata-driven methodology and workflow process for providing translational research informatics support. J Biomed Inform. 2009;42(2):377–81. 18929686 10.1016/j.jbi.2008.08.010 PMC2700030

[41] Harris PA, Taylor R, Minor BL, Elliott V, Fernandez M, O’Neal L, et al. The REDCap consortium: Building an international community of software platform partners. Journal of Biomedical Informatics. 2019;95:103208. 31078660 10.1016/j.jbi.2019.103208 PMC7254481

[42] National Committee for Quality Assurance. Initiation and Engagement of Substance Use Disorder Treatment (IET) [Internet]. [cited 2024 May 6]. Available from: https://www.ncqa.org/hedis/measures/initiation-and-engagement-of-substance-use-disorder-treatment/

[43] Harris AHS, Humphreys K, Bowe T, Tiet Q, Finney JW. Does meeting the HEDIS substance abuse treatment engagement criterion predict patient outcomes? J Behav Health Serv Res. 2010;37(1):25–39. 18770044 10.1007/s11414-008-9142-2

[44] National Committee for Quality Assurance. MY 2023 Quality Rating System (QRS) HEDIS Value Set Directory [Internet]. 2023 [cited 2024 May 6]. Available from: https://store.ncqa.org/my-2023-quality-rating-system-qrs-hedis-value-set-directory.html

[45] Ober AJ, Murray-Krezan C, Page K, Friedmann PD, Anderson J, Osilla KC, et al. Hospital addiction consultation service and opioid use disorder treatment: the START randomized clinical trial. JAMA Intern Med. 2025;185(6):624–33. 40193131 10.1001/jamainternmed.2024.8586 PMC11976642

[46] Anderson ES, Rusoja E, Luftig J, Ullal M, Shardha R, Schwimmer H, et al. Effectiveness of substance use navigation for emergency department patients with substance use disorders: an implementation study. Ann Emerg Med. 2023;81(3):297–308. 36402631 10.1016/j.annemergmed.2022.09.025

[47] Running Bear U, Poole EM, Muller C, Hanson JD, Noonan C, Trojan J, et al. The use of patient navigation to transition detoxification patients to substance use treatment in the Alaska Interior. Public Health in Practice. 2023;1(6):100418. 10.1016/j.puhip.2023.100418 PMC10448195 37635913

[48] Morgan KE, Forbes AB, Keogh RH, Jairath V, Kahan BC. Choosing appropriate analysis methods for cluster randomised cross-over trials with a binary outcome. Stat Med. 2017;36(2):318–33. 27680896 10.1002/sim.7137

[49] Arnup SJ, McKenzie JE, Hemming K, Pilcher D, Forbes AB. Understanding the cluster randomised crossover design: a graphical illustraton of the components of variation and a sample size tutorial. Trials. 2017;18(1):381. 28810895 10.1186/s13063-017-2113-2 PMC5557529

[50] Hemming K, Kasza J, Hooper R, Forbes A, Taljaard M. A tutorial on sample size calculation for multiple-period cluster randomized parallel, cross-over and stepped-wedge trials using the shiny CRT calculator. Int J Epidemiol. 2020;49(3):979–95. 32087011 10.1093/ije/dyz237 PMC7394950

[51] Turner RM, White IR, Croudace T, PIP Study Group. Analysis of cluster randomized cross-over trial data: a comparison of methods. Stat Med. 2007 Jan 30;26(2):274–89. 10.1002/sim.2537 16538700

[52] Li F, Forbes AB, Turner EL, Preisser JS. Power and sample size requirements for GEE analyses of cluster randomized crossover trials. Stat Med. 2019;38(4):636–49. 30298551 10.1002/sim.7995 PMC6461037

[53] Regression Models and Life-Tables [Internet]. Journal of the Royal Statistical Society: Series B (Methodological). 1972. p. 34 187–202. 10.1111/j.2517-6161.1972.tb00899.x

[54] Matson TE, Galea S. Understanding inequitable healthcare: Methodological approaches, challenges, and opportunities. Am J Epidemiol. 2024 Dec 11; kwae454. 10.1093/aje/kwae454 PMC13249008 39663411

[55] Smith JD, Li DH, Rafferty MR. The implementation research logic model: a method for planning, executing, reporting, and synthesizing implementation projects. Implement Sci. 2020;15(1):84. 32988389 10.1186/s13012-020-01041-8 PMC7523057

[56] Woodward EN, Matthieu MM, Uchendu US, Rogal S, Kirchner JE. The health equity implementation framework: proposal and preliminary study of hepatitis C virus treatment. Implement Sci. 2019;14(1):26. 30866982 10.1186/s13012-019-0861-y PMC6417278

[57] Proctor EK, Powell BJ, McMillen JC. Implementation strategies: recommendations for specifying and reporting. Implement Sci. 2013;8(1):139. 24289295 10.1186/1748-5908-8-139 PMC3882890

[58] Glasgow RE, Vogt TM, Boles SM. Evaluating the public health impact of health promotion interventions: the RE-AIM framework. Am J Public Health. 1999;89(9):1322–7. 10474547 10.2105/ajph.89.9.1322 PMC1508772

[59] Moullin JC, Dickson KS, Stadnick NA, Rabin B, Aarons GA. Systematic review of the exploration, preparation, implementation, sustainment (EPIS) framework. Implement Sci. 2019;14(1):1. 30611302 10.1186/s13012-018-0842-6 PMC6321673

[60] Aarons GA, Hurlburt M, Horwitz SM. Advancing a conceptual model of evidence-based practice implementation in public service sectors. Adm Policy Ment Health. 2011;38(1):4–23. 21197565 10.1007/s10488-010-0327-7 PMC3025110

[61] Aarons GA, Ehrhart MG, Farahnak LR, Sklar M. Aligning leadership across systems and organizations to develop a strategic climate for evidence-based practice implementation. Annu Rev Public Health. 2014;35:255–74. 24641560 10.1146/annurev-publhealth-032013-182447 PMC4348088

[62] Weiner BJ, Lewis CC, Stanick C, Powell BJ, Dorsey CN, Clary AS, et al. Psychometric assessment of three newly developed implementation outcome measures. Implement Sci. 2017;12(1):108. 28851459 10.1186/s13012-017-0635-3 PMC5576104

[63] Aarons GA, Ehrhart MG, Farahnak LR. The implementation leadership scale (ILS): development of a brief measure of unit level implementation leadership. Implement Sci. 2014;9(1):45. 24731295 10.1186/1748-5908-9-45 PMC4022333

[64] Ehrhart MG, Aarons GA, Farahnak LR. Assessing the organizational context for EBP implementation: the development and validity testing of the implementation climate scale (ICS). Implement Sci. 2014;9(23):157. 25338781 10.1186/s13012-014-0157-1 PMC4210525

[65] Chokron Garneau H, Cheng H, Kim J, Abdel Magid M, Chin-Purcell L, McGovern M. A Pragmatic Measure of Context at The Organizational Level: The Inventory Of Factors Affecting Successful Implementation And Sustainment (IFASIS). Implement Sci. In Press

[66] Chokron Garneau H, Cheng H, Kim J, Abdel Magid M, Chin-Purcell L, McGovern M. The Inventory of Factors Affecting Successful Implementation and Sustainment. [Internet]. The Center for Dissemination and Implementation at Stanford (C-DIAS); 2023 [cited 2025 Feb 26]. Available from: www.c-dias.org

[67] Saldana L. The stages of implementation completion for evidence-based practice: protocol for a mixed methods study. Implement Sci. 2014;9(1):43. 24708893 10.1186/1748-5908-9-43 PMC4234147

[68] Saldana L, Chamberlain P, Bradford WD, Campbell M, Landsverk J. The cost of implementing new strategies (coins): a method for mapping implementation resources using the stages of implementation completion. Child Youth Serv Rev. 2014;39(1):177–82. 24729650 10.1016/j.childyouth.2013.10.006 PMC3979632

[69] McBrien KA, Ivers N, Barnieh L, Bailey JJ, Lorenzetti DL, Nicholas D, et al. Patient navigators for people with chronic disease: a systematic review. PLoS One. 2018;13(2):e0191980. 29462179 10.1371/journal.pone.0191980 PMC5819768

[70] Brownson RC, Shelton RC, Geng EH, Glasgow RE. Revisiting concepts of evidence in implementation science. Implement Sci. 2022;17(1):26. 35413917 10.1186/s13012-022-01201-y PMC9004065

