# Supplementary figures and images for: Design of a cluster-randomized, hybrid type 1 effectiveness-implementation trial of a care navigation intervention to increase substance use disorder treatment engagement: study protocol
Source: Addict Sci Clin Pract. 2025 Oct 1;20:78. doi: 10.1186/s13722-025-00605-7 (PMC12486859; doi:10.1186/s13722-025-00605-7)

 
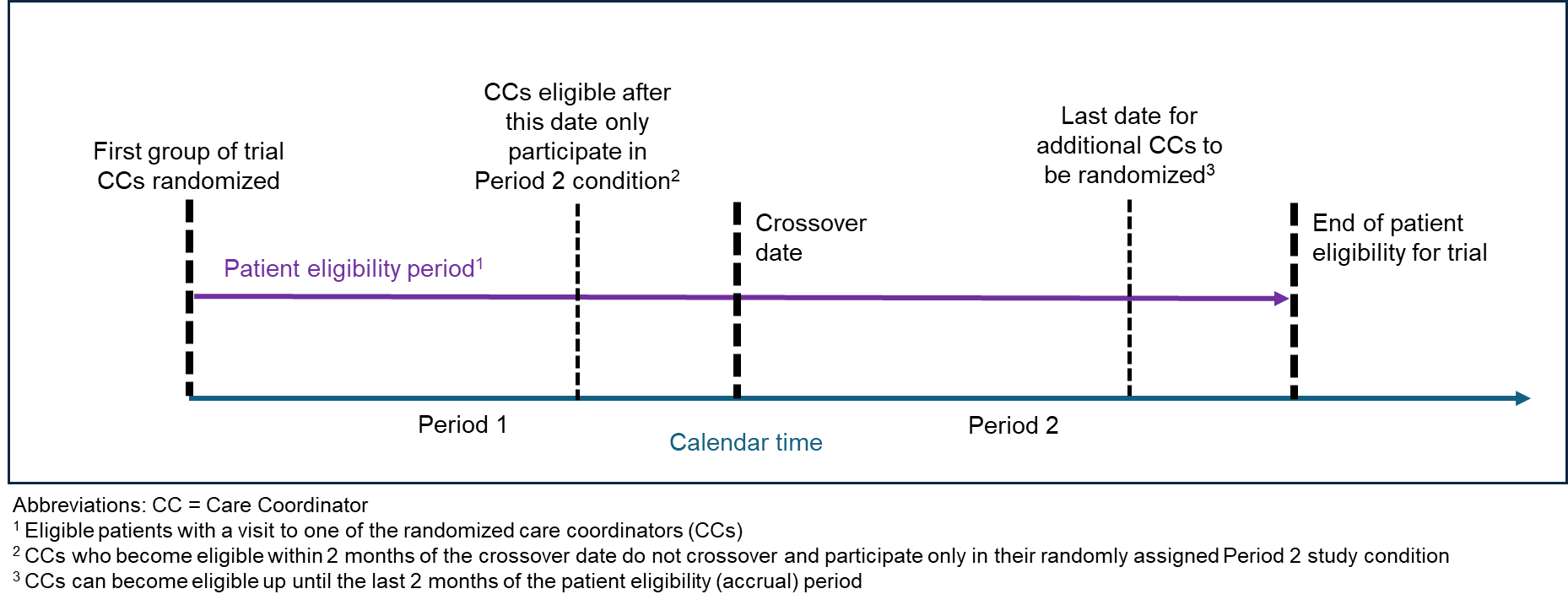

Supplement: Supplementary file 1 — Supplementary material 1: Time periods for mental health care coordinator (CC) and patient eligibility in the ABC-SUD trial. [file 13722_2025_605_MOESM1_ESM.docx]
