# Supplementary material for: Design of a cluster-randomized, hybrid type 1 effectiveness-implementation trial of a care navigation intervention to increase substance use disorder treatment engagement: study protocol
Source: Addict Sci Clin Pract. 2025 Oct 1;20:78. doi: 10.1186/s13722-025-00605-7 (PMC12486859; doi:10.1186/s13722-025-00605-7)
Supplement: Supplementary file 3 — Supplementary material 3: Details of the ABC-SUD pilot. [file 13722_2025_605_MOESM3_ESM.docx]

**Details of the ABC-SUD Pilot**

Mental health access center managers emailed staff and identified four care coordinator volunteers. We included care coordinators that had been employed for at least one month, had completed trainings relevant to their clinical role in the health system, and were scheduled to conduct assessments. All four eligible volunteers consented to participate over email. After revealing randomization assignments, the two intervention group care coordinators were instructed to refer all eligible patients to care navigators via a template programmed into the EHR. Eligible patients included adults ≥18 years of age who completed an SUD assessment and treatment planning visit from pilot study launch to completion (July 9, 2024 through November 27, 2024).

The study team met every two weeks with care coordinators and a manager to conduct plan-do-study-act cycles (24) to improve recruitment and intervention procedures. Meeting attendees shared patient stories, discussed care navigation cases, reviewed eligibility and recruitment reports, and identified and reviewed change targets. From September 16, 2024 through November 27, 2024, we modified patient eligibility criteria to evaluate feasibility and applicability of recruiting and conducting care navigation with patients with SUD seeking mental health treatment (i.e., adult patients with a documented SUD diagnosis on the day of their assessment and treatment planning visit for mental health care). As a result of the pilot, we modified recruitment scripts, adapted care coordinator training materials, solidified the cadence of care navigation outreach and visits, identified additional parameter estimates for the statistical power analyses to refine our estimates of the trial’s power, and confirmed we would maintain the original patient eligibility criteria for the trial.

Based on refined power estimates, we decided to switch from a parallel-group design to a crossover design to maximize statistical power and increase the ability to detect implementation changes. Additionally, before we launched the pilot, we had intended to employ a "vanguard" randomization (25), a type of stratified randomization in which pilot (or vanguard) care coordinators are pre-randomized to begin piloting the intervention but then retain their randomization status for the subsequent trial. The four vanguard clinicians recruited for the pilot study remain eligible for the trial because the crossover design has each clinician experience both treatment assignments. Specifically, the four vanguard clinicians are randomized to an allocation sequence for the trial, along with other eligible (non-vanguard) clinicians, as described above.
