# Supplementary material for: Design of a cluster-randomized, hybrid type 1 effectiveness-implementation trial of a care navigation intervention to increase substance use disorder treatment engagement: study protocol
Source: Addict Sci Clin Pract. 2025 Oct 1;20:78. doi: 10.1186/s13722-025-00605-7 (PMC12486859; doi:10.1186/s13722-025-00605-7)
Supplement: Supplementary file 4 — Supplementary material 4: Study information sheet used for verbal consent of patients for care navigation. [file 13722_2025_605_MOESM4_ESM.pdf]

KAISER PERMANENTE®
Kaiser Permanente Washington
Health Research Institute

ABC Study

Addressing Barriers to Care for Substance Use (ABC-SUD)
Study Information Sheet

RESEARCHERS
Principal Researcher: Joe Glass, PhD, MSW, 206-287-4266
Project Manager: Chay Davis, PhD, 206-714-4738

OVERVIEW
- We are asking you to be in a research study funded by the National Institutes of Health.
- Please take as much time as you need to read through this information.
- Ask questions if anything isn't clear or if you would like more information.
- You do not have to be in this study. If you do decide to take part, you can quit the study at any time.

WHAT IS THIS STUDY ABOUT?
This study is looking at whether offering a new service can help people engage in care offered by Kaiser Permanente for alcohol or drug use. We call this service "care navigation." We are contacting you because a clinician at Kaiser Permanente offered this service to you. This study is part of a collaboration between Kaiser Permanente Washington Health Research Institute and Stanford University. It is funded by the National Institutes of Health. We anticipate approximately 400 participants will be in the study.

WHAT WILL HAPPEN IF I TAKE PART IN THIS STUDY?

- **Meet with a care navigator** on the study team by phone over the course of approximately 7 weeks. Calls will be scheduled between you and your navigator during times that are convenient for you.
- **Agree to speak with the care navigator** about your care for substance use.
- **Let us collect some information from your Kaiser Permanente medical record** about health care provided to you from January 1, 2023, to December 31, 2026. We will use a computer to collect information about your age, gender, racial and ethnic background, the neighborhood you live in, and the health services you use. Sensitive data and/or records on your use of alcohol, drugs, and/or other addictive products, disease history, doctor's visits, mental health diagnoses, and the results of your assessments for behavioral health and alcohol and substance use will also be collected. All of this sensitive information will be stored separately from information that identifies you, such as your name and phone number. Some specific information discussed with Kaiser Permanente, namely the treatment plan developed for you, and some of the information discussed with care navigators, such as the barriers you encounter and what the care navigator did, will be collected and used as part of the study.

DURING YOUR MEETINGS WITH THE CARE NAVIGATOR, they will:

- Review the treatment plan you and your clinician discussed during the visit in which you were initially offered care navigation.
- Talk about challenges you may face while starting/ receiving treatment and ways to overcome them.
- Discuss your treatment progress and next steps in treatment.

WILL THERE BE ANY COST TO ME?
The visits with the care navigator are at no cost to you. Any other care you get will still be billed to you or your insurance, with any required copays, just as it usually would.

WILL BEING IN THIS STUDY HELP ME?
This study may help you start and continue substance use treatment. We hope the results of this study will help improve medical care in the future for people who use substances.

CAN ANYTHING BAD HAPPEN TO ME FROM BEING IN THIS STUDY?
While the program is meant to help you connect to effective care that interests you, there could be risks which are not currently known. It is also possible that someone other than the researchers could see your private study information, but we take many steps, outlined in the next section, to prevent that.

HOW WILL YOU PROTECT MY PRIVACY?

- The researchers listed on the first page and the study staff will use your information for research only.
- You are assigned a unique study ID number that is used in study records. Only study team members can link your study ID number to your medical record and contact information.
- Some information we collect for the study is stored outside of your medical record. Data is stored electronically on password-protected files at the offices of Kaiser Permanente Washington Health Research Institute. We will store this data separately from information that identifies you, such as your name, email, and phone number.
- Both your medical record and study records are protected by state and federal privacy laws, such as "HIPAA" (see below).
- Research data that does not identify you will be shared with study team members at Stanford University and with our study funder.
- To maximize the benefits of public health research, our funder, the National Institutes of Health, requests that research data be made available for other researchers to use. Thus, summary data from focus groups will be posted to a public scientific data sharing archive. We will not post any information that could identify you such as your name, address, or birth date, so it is unlikely that someone would know the information came from you. However, it may be possible to identify you by connecting the information in the database with other public information (including information you tell people or post about yourself). The risk of this happening is currently low, but it may become more likely in the future.

HOW DOES HIPAA APPLY TO THIS STUDY?
Your health information is protected by a federal privacy law called HIPAA, or the Health Insurance Portability and Accountability Act of 1996. Kaiser Permanente and Stanford University must follow this privacy law. According to HIPAA, the information collected for this study is part of that protected health information. HIPAA requires that we tell you the following:

By participating in this study, you are giving Kaiser Permanente permission to allow the researchers to collect, use, and share the following information about you for this study:

1. Your interview responses
2. Your medical record information as described above

We will do our best to protect your confidentiality by using standard security measures as required by law. We will also remove or separate information that identifies you (such as your name or address) from the rest of your health information whenever possible. Everyone at Kaiser Permanente with access to your information has received training in the protection of sensitive information. Still, there is a small chance your information could be released accidentally. There are also certain situations where we may be required by law to release your information. People overseeing the research may also be allowed to review and copy information in your records related to this study. Once your information has been given to others, it may no longer be protected by state or federal privacy laws. It will be

protected by other rules and agreements with the recipients. However, there is still a risk that a recipient could share your information without your permission.

To be in the study, you must agree to this use of your health information. This permission for the researchers to retain your health information for this study ends on May 31, 2032.

DO I HAVE TO BE IN THIS STUDY?
No, being in this study is up to you. You are free to say no now or to leave the study at any time later. Either way, there will be no penalty. Your decision won't affect the health care you receive or the benefits that you are entitled to. You can get care, treatment, and testing for substance use without being in this study.

WHAT HAPPENS IF I SAY YES, BUT CHANGE MY MIND LATER?
You may change your mind at any time about letting us use your information for this study. If you change your mind, you may take back your consent by sending an email to the project manager, Chay Davis, at Chay.J.Davis@kp.org. If you take back your consent, it will not affect your benefits at Kaiser Permanente. We may still use the study information we collected before we received your email taking back your consent, but we will destroy any record of your name or other information that could identify you.

WHO SHOULD I CONTACT WITH MEDICAL QUESTIONS OR CONCERNS?
You should contact your regular medical provider(s) for questions or concerns as you normally would. If you take part in the intervention, the care navigator will work closely with your regular medical team and may reach out to follow up on care you receive from your team.

WHO SHOULD I CONTACT ABOUT THE STUDY?
If you have questions, concerns, or complaints about the study, please contact the lead researcher at the telephone numbers listed on the first page of this form or email the project manager, Chay Davis, at Chay.J.Davis@kp.org.

This research is overseen by an Institutional Review Board (IRB). An IRB is a group of people who perform independent review of research studies. You may talk to them at (951) 739-6781 or email KPInterregionalIRB@kp.org if:

- You have questions, concerns, or complaints that are not being answered by the research team.
- You are not getting answers from the research team.
- You cannot reach the research team.
- You want to talk to someone else about the research.
- You have questions about your rights as a research subject.
