# Supplementary material for: Design of a cluster-randomized, hybrid type 1 effectiveness-implementation trial of a care navigation intervention to increase substance use disorder treatment engagement: study protocol
Source: Addict Sci Clin Pract. 2025 Oct 1;20:78. doi: 10.1186/s13722-025-00605-7 (PMC12486859; doi:10.1186/s13722-025-00605-7)
Supplement: Supplementary file 5 — Supplementary material 5: Map of patient needs/barriers expressed by people seeking substance use disorder treatment to the core functions of care navigation and pre-specified intervention forms. [file 13722_2025_605_MOESM5_ESM.docx]

| **Additional File 5.** Map of patient needs/barriers expressed by people seeking substance use disorder treatment to the core functions of care navigation and pre-specified intervention forms. | | | | |
| --- | --- | --- | --- | --- |
| **Need/Barrier** | **Example Need/Barriers** | **Core Functions**  **(what we are trying to do)** | **Pre-specified Intervention Forms**  **(ways to accomplish the core function)** | **Example Ways to Deliver Intervention Forms** |
| **Health or mental health needs** | - Unmanaged chronic medical or mental health condition - Medication needs (e.g., refill) | - Promote whole health while addressing substance use | - Facilitate communication with health or mental health clinicians - Empower patients to communicate needs to health care clinicians - Offer resources that address health or mental health needs - Help patient balance healthcare priorities | - Warm-handoff patient to appropriate facilities/representatives - Use complex reflections to summarize patient’s priorities - Query extremes - Double-sided reflection - Ask for elaboration/examples - Look back or look forward - Notice goals and values |
| **Varying motivation for SUD treatment** | - Feels like current gains or attempts to cut back have been sufficient - Getting into treatment seems too hard - Low problem recognition - Wants to stop without treatment | - Boost motivation for SUD treatment | - Use motivational interviewing skills, strategies, and spirit to direct conversations to address ambivalence about treatment - Identify and reflect values that you have heard to affirm and reinforce reasons for change | - Query patient’s original reasons for seeking treatment, amplify patient’s change talk - Affirm steps taken so far - Employ appropriate MI micro-skills (e.g., double-sided reflections; complex reflections; respond to sustain talk [e.g., Overshooting reflection, reframe]) - Make a plan for regular contact while patient seeks, initiates, and engages in treatment - Accommodate patient preferences - Emphasize patient autonomy |
| **Personal belief or values that conflict with treatment** | - Fears or negative perceptions about treatment - Doesn’t like answering personal questions - Low self-efficacy/confidence - Believes they can’t change - Other beliefs driven by social or internalized substance use stigma (e.g., treatment is for “alcoholics”/not for me, doesn’t like the “types of people” in treatment, treatment doesn’t work, etc.) | - Reconcile beliefs or values that conflict with treatment | - Use motivational interviewing skills, strategies, and spirit to direct conversations to address ambivalence about treatment - Seek to understand patient beliefs about treatment - Understand and reflect patient values to affirm and reinforce reasons for change - Validate concerns without developing sustain talk further - Check and help patient adjust their treatment plan so it remains optimized to patient situation, goals, and values | - Use simple reflections with sustain talk - Use complex reflections with change talk - Double-sided reflections, ending on change talk - Elicit-provide-elicit: ask permission to provide input, offer information, ask patient what they think - Emphasize patient autonomy. Partner with patient to explore possible solutions and/or alternatives to stay engaged with any treatment/change effort. Reinforce any positive change efforts. - Normalize that undertaking change can feel uncomfortable - Engage patient to understand their perspectives on treatment. Listen and reflect. - Avoid pressuring patient, emphasize patient’s autonomy. - Stay alert for opportunities to evoke change talk |
| **Stigma or privacy concerns** | - Worried people at work might find out they are getting treatment - Fears judgment by others | - Balance tradeoffs of privacy/stigma and getting treatment | - Elicit patient beliefs and expectations about treatment - Reflect patient concerns, facilitate patient balancing priorities of privacy against values and need for treatment | - Open-ended questions to explore concerns - Offer information if patient is misinformed or doesn’t have complete information. Use elicit-provide-elicit strategy. - Reflect patient values and priorities - Pros and cons list - Use importance rulers |
| **Patient resource or time constraints** | - Can’t afford treatment/copays - Caregiving responsibilities - Doesn’t have a way to get to treatment - Caregiving responsibilities - Hasn’t found time to call the treatment center - Too busy at work or in life to add another thing - Can’t take time off work to attend treatment program - Incompatible schedule (e.g., only available in evenings and treatment is in the day) - Lack of private space for telehealth meeting - Unfamiliar with using apps or technology recommended by SUD counselor, hesitant to explore them - Does not have reliable internet connection or equipment (computer, phone) | - Overcome patient constraints by problem solving, sparking creativity, and confidence | - Elicit constraints that pose barriers to care - Empower patients to communicate needs to health care clinicians - Problem-solve ways to overcome barriers - Check that treatment plan remains optimized to patient situation and adjust and adapt as necessary - Facilitate assistance from health care system and community agencies (e.g., care coordinators, resource specialists, treatment agencies) - Provide digital literacy or digital access assistance - Employ motivational interviewing OARS skills to help focus the client on the change goal despite barriers | - Link patient to resources to address barriers (e.g., resource specialists, appointment transportation, loaner smartphone) - Help patient "walk through" technology required for treatment - Explore alternative or additional treatment options (e.g., virtual, asynchronous, alternate appointment times) - Explore the tension between patient constraints and the change goal by using importance rulers, reflecting and asking for elaboration, but guide the conversation to stay with the change goal, or identify a new one if needed |
| **Cultural or equity concerns** | - Language barrier - Cultural mismatch with treatment philosophy, interventions, or clinicians - Wants to be counseled by someone who is in recovery - Worries about being treated differently because of their race/ethnicity | - Support culturally responsive treatment | - Seek to understand patient cultural concerns, check for accurate understanding - Empower patient to select a treatment that aligns with their personal and cultural values - Accommodate patient preferences (e.g., preferred method of communication) - Seek to understand and if needed, improve patient’s relationship with health care systems (e.g., collect questions, educate) | - Help patient adjust their treatment plan so it remains optimized to patient situation, goals, and values - Offer translation or interpreter resources, if available - Reflect conflicting values and priorities - Match patient to clinician of similar culture and/or lived experience - Emphasize autonomy - Help patient seek support resources from community |
| **Health care system limitations and complexity** | - Delays due to required intake paperwork - Waiting for insurance authorization - Confused about who they are supposed to call next - No space available/not taking new patients - Specific modality of desired treatment is limited/no clinician available - Long wait list for preferred treatment option | - Understand, problem-solve, and/or plan to overcome health system limitations and complexities | - Seek to understand patient’s experience of and perspectives on health care system complexities - Spark creativity, confidence, problem solving, and/or provide information to overcome system limitations and complexities - Empower patient to communicate and advocate for their needs with health care system/clinician - Maintain regular contact while patient seeks, initiates, and engages in treatment | - Partner with patient to gather, organize information (e.g., finding agencies, handling administrative issues, remembering appointments) - Offer follow-up after scheduled visit or on a timeline that is customized to patient needs - Help patient identify necessary contact information, complete warm handoff when possible - Demonstrate understanding, reflect, and validate patient experience then guide the conversation to stay with the change goal, or identify a new one if needed |
| **Lack of social support for treatment** | - Lack of partner, friends, or others who are a strong source of support for treatment - Social circles primarily engage in substance-centered environments (i.e. at bars) | - Bolster social support for treatment | - Reflect the social support-related conflict/difficulty but guide the conversation to stay with the treatment/change goal - Elicit patient’s understanding/perspective about social support for treatment - Problem-solve ways to overcome social barriers | - Encourage new opportunities for social support (e.g., finding peer support groups) - Explore additional social support needs for treatment - Use OARS skills to build patient interest to find and reinforce their social support systems; use affirmations to reinforce change efforts and change talk |
