# Supplementary material for: Design of a cluster-randomized, hybrid type 1 effectiveness-implementation trial of a care navigation intervention to increase substance use disorder treatment engagement: study protocol
Source: Addict Sci Clin Pract. 2025 Oct 1;20:78. doi: 10.1186/s13722-025-00605-7 (PMC12486859; doi:10.1186/s13722-025-00605-7)
Supplement: Supplementary file 6 — Supplementary material 6: Statistical Analysis Plan. [file 13722_2025_605_MOESM6_ESM.pdf]

ABC-SUD Trial

Statistical Analysis Plan (SAP)

Version 2.0

December 6, 2024

Table of Contents

LIST OF ABBREVIATIONS **3**

OVERVIEW OF CHANGES FROM PRIOR VERSION OF SAP **4**

1.0 SUMMARY OF STUDY DESIGN AND PROCESSES **5**

1.1 Study Objectives 5
1.2 Study Design and Intervention Groups 5
1.3 Randomization 5
1.4 Sample and Sample Size 6

2.0 GENERAL PROCEDURES AND DEFINITIONS **8**

2.1 Intent-to-treat (ITT) Analysis 8
2.2 Time Periods Anchored on Randomization 8
2.2.1 Trial Start Date 8
2.2.2 Crossover Date and Periods 8
2.2.3 Trial Patient Eligibility Period 8
2.3 Time Periods Anchored on Patient Visits 8
2.3.1 Index Visit 8
2.3.2 Follow-up Period 8
2.4 Pilot Activities 8
2.4.1 Data-Only Pilot 8
2.4.2 Trial Pilot 9
2.5 Multi-Phase SAP Development Process 9

3.0 STUDY POPULATION **10**

3.1 Assignment of Patients to Care Coordinators 10

4.0 OUTCOME MEASURES **11**

5.0 ANALYSES OF OUTCOME MEASURES **12**

5.1 Analysis of Primary Outcome Measure 12
5.2 Analysis of Secondary and Additional Outcome Measures 12

6.0 POWER CONSIDERATIONS **13**

7.0 DESCRIPTIVE ANALYSES **15**

7.1 Analyses of Demographic and Baseline Data 15
7.1 Crossover 15

8.0 OTHER CONSIDERATIONS **16**

8.1 Missing Data 16

9.0 SAFETY AND INTERIM ANALYSES **17**

REFERENCES **18**

LIST OF ABBREVIATIONS

ABC-SUD	Addressing Barriers to Care for Substance Use Disorder Trial
CC	Care Coordinator
CRXO	Cluster Randomized Crossover
EHR	Electronic Health Record
IRB	Institutional Review Board
ITT	Intent-To-Treat
KPWA	Kaiser Permanente Washington
MDD	Minimal Detectable Difference
SAP	Statistical Analysis Plan
SUD	Substance Use Disorder

OVERVIEW OF CHANGES FROM PRIOR VERSION OF SAP

The prior version of the SAP was Version 1.0 (v1.0), which was completed prior to the launch of the trial pilot. SAP v1.0 described the anticipated trial design, which was originally planned to be a parallel group, cluster randomized trial. In addition, v1.0 described the preliminary plan for the trial sample and primary outcome definition, with the intention that analyses of pilot data would inform the final specification of the trial design and primary outcome definition. Based on analyses of the pilot data and discussion with study co-investigators and health system partners, we decided to convert our study from a parallel group cluster-randomized design into a cluster-randomized crossover (CRXO) design. As compared to the prior parallel group design, the CRXO design increased the study's power and provides greater control for potential imbalance in cluster-level baseline covariates (because clusters are observed under both intervention and control conditions under the crossover design). This SAP version (Version 2.0) is completed prior to the trial launch and specifies the final trial design, including eligibility criteria and primary outcome definition.

1.0 SUMMARY OF STUDY DESIGN AND PROCESSES

1.1 Study Objectives

The Addressing Barriers to Care for Substance Use Disorder (ABC-SUD) study seeks to evaluate the effectiveness of a care navigation intervention within a mental health access center while generating information about how to optimally implement care navigation in routine clinical care.

This Statistical Analysis Plan (SAP) addresses the following study aim: to evaluate the effect of care navigation on increasing engagement in SUD treatment among patients who contact a mental health access center to seek care for SUD.

The ABC-SUD study also includes implementation science goals. We will describe the implementation of care navigation and examine how perceptions about the context and the innovation impacts implementation of care navigation. The implementation science component of this study is beyond the scope of this SAP and will be described in the ABC-SUD study protocol manuscript.

1.2 Study Design and Intervention Groups

The ABC-SUD study is a cluster-randomized crossover (CRXO) trial, with care coordinators as the unit of randomization. The crossover design will be conducted over two periods (of approximately 6-7 months per period, referred to as Period 1 and Period 2), with clusters (care coordinators) randomized to one of the following two sequences: usual care (also referred to as service as usual) followed by the intervention condition or intervention followed by usual care (see **Figure 1.2**).

Figure 1.2 Two-period cluster randomized crossover trial

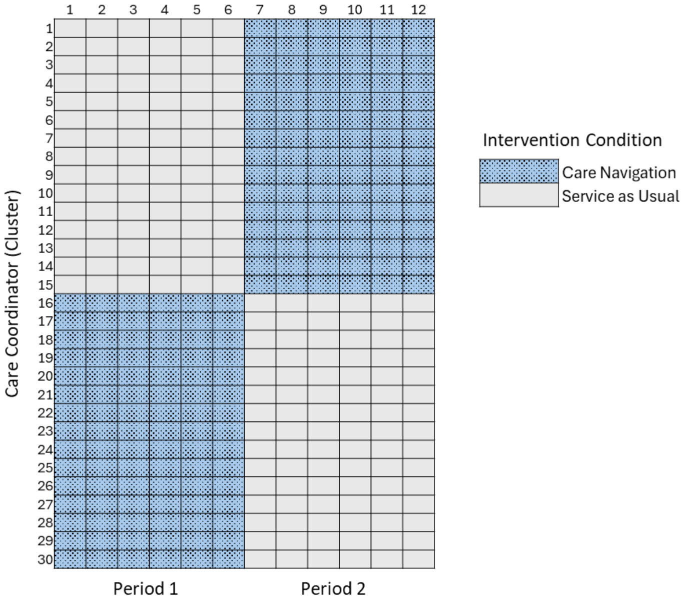

The intervention being tested is a novel care navigation intervention for patients seeking SUD treatment who contact care coordinators (also referred to in this SAP as "providers" or "clinicians") in a mental health access center. The control condition is usual care offered by care coordinators. Details on the intervention conditions are included in the study protocol manuscript. Cluster-randomization is at the clinician (care coordinator) level because care delivery leaders deemed that it is infeasible for any single care coordinator to offer care navigation to some patients and not others within a given time period. We anticipate that 30-40 care coordinators will be randomized.

The CRXO design is reasonable given that we do not expect carryover of the intervention, because the care navigation intervention is administered by care navigators external to the mental health access center, and because the ability of care navigators to offer care navigation is controlled by prompts embedded within the electronic health record (EHR) that can be turned on or off easily by the health system.

1.3 Randomization

We will randomize eligible mental health care coordinators 1:1 to the two allocation sequences—defined by which of the two intervention conditions is provided first versus second—using a computer-generated list of random numbers created by the study biostatistician. Given the

crossover design, whereby providers will receive both intervention conditions in a random order, baseline clinician covariates are naturally balanced across intervention conditions (provided clinicians remain in the study for the full follow-up period). We therefore do not plan to stratify the randomization on baseline clinician-level covariates; the potential for imbalance due to possible clinician drop-out is described below in the missing data section. We note that the 4 “vanguard” clinicians recruited to participate in the pilot study (of whom 2 were randomized to pilot the care navigation intervention) will be re-randomized for the trial. As described above, we do not expect carryover of the intervention, and given the crossover design, these providers will experience both intervention conditions.

Care coordinators will become enrolled in the study and randomized to one of the two allocation sequences as they become eligible (see care coordinator eligibility in Section 1.4). So that allocations will be approximately balanced over time, we will employ a permuted block randomization using variable (random) block sizes of 2 and 4. Group assignments will be concealed in a password protected file by the biostatistical team until coordinators become eligible.

We anticipate that most care coordinators will be randomized within a 1-month period of the initial randomization date. However, to maximize the study sample, we will randomize additional care coordinators who become eligible later, for example if they are newly hired. For providers who become eligible after the trial has launched, whether they will experience both intervention conditions will depend on the timing of their eligibility and randomization date (see **Figure 1.3**). If they become eligible with at least $M = 2$ months left within Period 1, they will cross over between both intervention conditions; if they become eligible with less than M months before the end of Period 1, they will not cross over and will only experience the Period 2 intervention assignment (beginning on the crossover date); eligibility ends if less than M months remain before the end of Period 2 (end of the trial).

Figure 1.3. Time periods for care coordinator (CC) and patient eligibility in the ABC-SUD trial

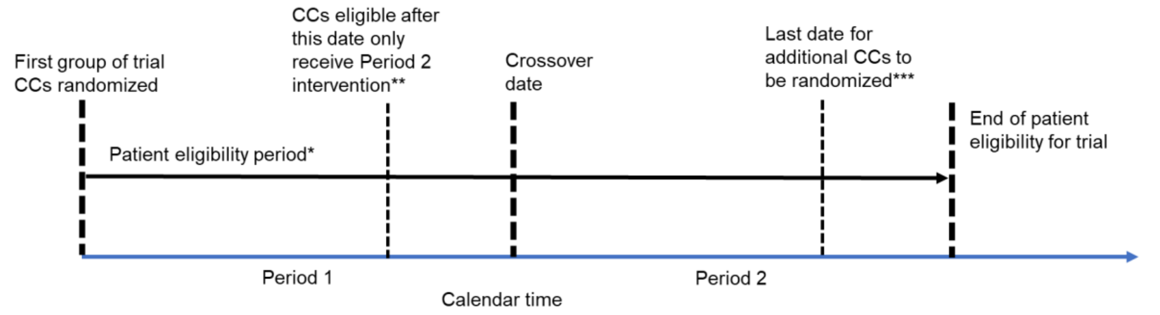

*Eligible patients with a visit to one of the randomized care coordinators (CCs)
**CCs who become eligible within M months of the crossover date do not crossover and receive only the Period 2 intervention
***CCs can become eligible up until the last M months of the patient eligibility (accrual) period (see Section 1.3)

1.4 Sample and Sample Size

Care coordinators are eligible if they conduct video or phone-based assessment and treatment planning visits, have been employed for at least 1 month, have completed trainings related to their clinical role in the health system, and are scheduled to conduct assessments. To maximize the number of care coordinators in the study, care coordinators could become eligible if they meet criteria at any time between the trial start date (see Section 2.2.1) and a certain number of months (denoted by M) prior to the end of the patient eligibility period (to allow at least M months of patient accrual; see **Figure 1.3**). Using preliminary data, we expect a sample size of approximately 30-40 care coordinators.

The sample of patients eligible for the trial includes individuals with a visit to a randomized care coordinator during the patient eligibility period (defined in Section 2.2.3) who are ≥18 years of age, have an assessment visit with a care coordinator, and receive an SUD treatment plan.

2.0 GENERAL PROCEDURES AND DEFINITIONS

2.1 Intent-to-treat (ITT) Analysis

Unless otherwise specified, all analyses will follow an intent-to-treat principle whereby care coordinators will be analyzed according to the intervention arm to which they were randomized regardless of the subsequent sequence of events.

2.2 Time Periods Anchored on Randomization

2.2.1 Trial Start Date

The trial start date is the date that the first patient is enrolled in the trial. Thus, the trial start date is equivalent to the first day of the trial patient eligibility period (see Section 2.2.3 below).

2.2.2 Crossover Date and Periods

The crossover date is initially planned to be 6 months after trial start; however, the rate of patient accrual will be monitored, and the crossover date may be extended to 7 months after trial start to meet patient accrual goals (180 patients per Period, assuming 30 providers with an average of 6 patients per Period). Period 1 is defined as the period from trial start until the day before the crossover date. Period 2 is defined as the period from the crossover date, through the end of the trial. The length of Period 2 is initially planned to be the same as the length of Period 1, but it may be extended up to a maximum of 7 months to meet patient accrual goals, as described above. For instance, Period 2 could be longer than Period 1 if enrollment decreases during Period 2.

2.2.3 Trial Patient Eligibility Period

This is the period in which patients become eligible for the trial (i.e., for inclusion in the analytic sample). The patient eligibility period begins when the EHR prompt for care navigation is turned on for a care coordinator, which is anticipated to occur within a week of the care coordinator's randomization date. The patient eligibility period for all care coordinators ends 12-14 months following trial start, depending on the length of Period 1 and Period 2 defined above.

2.3 Time Periods Anchored on Patient Visits

2.3.1 Index Visit

This is the first eligible visit during the patient eligibility period (defined above), also referred to as the "assessment" visit. The approach to handling multiple eligible visits is described below in Section 3.1.

2.3.2 Follow-up Period

This is the period from the day of the index visit up to the end of follow-up for the primary outcome and each secondary outcome (defined in Section 4.0).

2.4 Pilot Activities

2.4.1 Data-Only Pilot

A secondary dataset—with data from before the start of the pilot and trial—was used to inform the trial design. This retrospective cohort included patients with a visit, at which they were ≥18 years, to one of the care coordinators at the mental health access center during the period from November 1, 2022 to October 31, 2023 (ending before any care coordinators were randomized for the ABC-SUD pilot or trial). Specific goals of these pilot analyses included:

- Assess feasibility of sample identification and outcome capture
- Estimate statistical power for the trial

2.4.2 Trial Pilot

Departmental managers identified 4 care coordinators who volunteered to participate in piloting the care navigator intervention. To pilot randomization procedures, the study biostatistician randomized 2 of these 4 care coordinators to pilot the care navigation intervention (the other 2 care coordinators continued with usual care). Specific goals are to a) confirm patient eligibility criteria, b) confirm selection of the primary and secondary outcomes and the approach to measuring them using electronic health record data, c) confirm the feasibility and acceptability of randomization procedures, d) evaluate the feasibility of care navigation protocol delivery by care coordinators, and e) evaluate feasibility of electronic health record templates to assist care coordinators when referring patients to care navigators. The pilot's recruitment goal is to offer care navigation to patients until a total of 10 patients agree to care navigation. We will conduct post-intervention quality improvement interviews with enrolled patients and employ user-centered design methods to obtain feedback on intervention design to inform changes. We will also develop processes for ascertaining participant data from the EHR. The patient eligibility period for the pilot is distinct from the patient eligibility period for the trial, with the trial launching after the pilot's accrual period is complete.

2.5 Multi-Phase SAP Development Process

Given the pragmatic nature of the trial, we develop the SAP in phases. The first version of the SAP (v1.0) was completed prior to the pilot and described preliminary specifications for the trial. As part of SAP v1.0, we planned to conduct analyses of the Data-Only Pilot (see Section 2.4.1) to inform modifications to the trial design following the pilot, including the randomization approach and specifications of the trial duration, primary outcome, and analysis approach. The second version of the SAP (v2.0) is completed prior to the trial launch and finalizes the specification of the trial design, including the primary outcome and analysis approach, along with descriptions of secondary outcomes. As needed, a final version of the SAP may subsequently be developed that includes any additional exploratory outcome measures and analyses, descriptive analyses, and sensitivity analyses.

3.0 STUDY POPULATION

Patients are eligible for inclusion in the trial sample if:

1) Have a visit with one of the ABC-SUD trial care coordinators during the patient eligibility period (defined above),
2) Adult aged 18 years or older at time of visit
3) Have an assessment visit with a trial care coordinator with a resulting SUD treatment plan

Visits meeting the above criteria are referred to as "eligible visits". Patients who have requested through the health system to opt out of research contact or chart review are excluded from the study sample. In the unlikely occurrence that a patient has a visit to a care coordinator but is not enrolled in the health plan (either KPWA or Medicaid, via KPWA's contract with Molina), the patient will be excluded (due to incomplete capture of covariate and outcome information; see Section 8.1). We will use administrative data to identify assessment visits (i.e., from assessment template IDs) and SUD treatment plans (i.e., SmartLists).

3.1 Assignment of Patients to Care Coordinators

If patients have eligible visits to multiple randomized care coordinators during the eligibility period, we plan to "assign" the patient to the care coordinator where they have their first eligible visit. This visit is referred to as the "index visit" (see Section 2.3.1). Based on preliminary data from our Data-Only Pilot (Section 2.4.1), the number of patients becoming eligible more than once over a 12-month period was small (<2% of eligible patients). Crossover is discussed in Section 7.1.

4.0 OUTCOME MEASURES

The *primary outcome* is a binary measure of treatment engagement, defined as completing ≥3 treatment visits for SUD within 48 days of the index visit (patient-level outcome). Treatment visits include medications, behavioral health visits in primary care, visits in specialty settings or with addiction specialists and could occur within Kaiser Permanente's clinics (e.g., the Addiction Recovery Service) or the contracted care network.

The secondary outcome is initiation of SUD treatment, defined as completing ≥1 treatment visits for SUD within 30 days of their index visit.

Additional outcomes include measures of treatment initiation (≥1 visit) considering 1) different time frames (i.e., 14, 60, and 90 of an index visit) and 2) treatment of SUD sequelae (i.e., including detoxification, primary care or urgent care medical evaluations for withdrawal, crisis services, etc.).

Table 4.0 Primary, secondary, and additional outcomes

Outcome	Definition
**Primary outcome**	
SUD treatment engagement*	Completion of ≥3 SUD treatment visits within 48 days of index visit (binary measure)
**Secondary outcomes**	
SUD treatment initiation in 30 days	Completion of ≥1 SUD treatment visit within 30 days of index visit (binary measure)
**Additional outcomes**	
SUD treatment initiation in 14 days*	Completion of ≥1 SUD treatment visit within 14 days of index visit (binary measure)
Any SUD care in 14 days	Completion of ≥1 SUD treatment visit and/or ≥1 visit treating SUD sequelae within 14 days of index visit (binary measure)
Any SUD care in 30 days	Completion of ≥1 SUD treatment visit and/or ≥1 visit treating SUD sequelae within 30 days of index visit (binary measure)
SUD treatment initiation in 60 days	Completion of ≥1 SUD treatment visit within 60 days of index visit (binary measure)
Any SUD care in 60 days	Completion of ≥1 SUD treatment visit and/or ≥1 visit treating SUD sequelae within 60 days of index visit (binary measure)
SUD treatment initiation in 90 days	Completion of ≥1 SUD treatment visit within 90 days of index visit (binary measure)
Any SUD care in 90 days	Completion of ≥1 SUD treatment visit and/or ≥1 visit treating SUD sequelae within 90 days of index visit (binary measure)

*Outcome time frames consistent with the Healthcare Effectiveness Data and Information Set.[1]

Exploratory analyses, time permitting, will examine time until SUD treatment initiation, reductions in substance use (for whom we won't have complete capture), and stratifying analyses across subgroups to understand potential inequities.

Implementation outcomes are outside the scope of this SAP and will be described in the ABC-SUD study protocol. Implementation outcomes are operationalized among care coordinators and patients, care navigators, and within the mental health access center as a whole. They include measures of reach, adoption, fidelity, competency, communication, and maintenance. Additional measures of the implementation context will be described.

5.0 ANALYSES OF OUTCOME MEASURES

5.1 Analysis of Primary Outcome Measure

For the primary patient-level binary outcome of SUD treatment engagement, we will apply a generalized linear mixed-effects model (GLMM). To account for clustering of patients within care coordinators, the model will include random intercepts for both cluster (care coordinator) and cluster-period.[2] Including both kinds of random intercept in the model allows for the possibility that individuals visiting the same provider in the same period (either Period 1 or Period 2) may be more strongly correlated than patients visiting the same provider but in different periods.[3] In addition, the model will include a main effect for Period to allow for secular trends in the outcome over time.[2] Specifically, our model will be of the form

$$logit(p_{itj}) = \alpha_0 + \alpha_1 t + \beta \cdot Int_{it} + u_i + u_{it},$$

where p_{itj} is the probability of the outcome for person j visiting care coordinator i during intervention period t (where t is a binary variable with $t = 0$ representing Period 1 and $t = 1$ representing Period 2); Int_{it} is an indicator for whether the care coordinator was in the intervention versus usual care condition during period t; and $u_i \sim N(0, \tau_B^2)$ and $u_{it} \sim N(0, \tau_W^2)$ are the cluster-specific and cluster-period random intercepts, both assumed to be normally distributed.

Rationale for model choice. The above statistical model was selected after reviewing relevant literature on analytic methods for CRXO studies.[2–7] In a simulation study focusing on binary outcomes, Morgan et al.[3] found that the above GLMM method (which they referred to as the "random-random" model) performed well but could have elevated type I error rates for studies with a small number of clusters if there was additional correlation of patients within the same cluster-period (i.e., corresponding to random effect variance $\tau_W^2 > 0$ using the above notation).[3] In contrast, conducting an aggregate data analysis, by analyzing the proportion of patients with the outcome in a cluster-period using a linear regression, had the correct type I error rates; however, power was considerably lower than the random-random method (Figure 5 in their paper).[3] A generalized estimating equations (GEE) approach was found to perform well but this method assumed balanced cluster-sizes across providers and periods.[7] Based on these studies and preliminary data indicating small extra-within period correlation of our outcome (i.e., τ_W^2 was estimated to be 0; see section on Statistical Power), we selected the above random-random GLMM. However, we note that our setting with a small expected cluster-period size (of ~6 patients per 6-month period) and moderate number of clusters (30-40) was not considered by these prior papers, nor was the setting where clusters may drop out or enter the study at different times. Therefore, time-permitting we will conduct a simulation study tailored to our trial setting and may modify the analytic approach if we find a better option (e.g., one that achieves the correct type I error without major sacrifices to power); any changes to the analytic approach will be specified in a subsequent version of the SAP (see Section 2.5) and finalized prior to collecting any outcome data on our trial sample.

5.2 Analysis of Secondary and Additional Outcome Measures

Secondary and additional outcomes are also binary measures and will be analyzed using the same analytic approach as the primary outcome.

6.0 POWER CONSIDERATIONS

Sample size and parameter estimates used for power calculations were estimated from preliminary data from the mental health access center from the Data-Only pilot conducted before the trial (Section 2.4.1). Over a 12-month accrual period, the average (SD) number of patients meeting eligibility criteria was 9.8 (8.2), corresponding to a coefficient of variation (CV) of cluster sizes equal to 0.84. Given subsequent efforts within the access center to standardize the SUD assessment process prior to the trial, as well as the option for our trial period to extend to 14 months, we used an average cluster size of 12 (corresponding to an average of 6 patients per provider in each intervention period).

Intraclass correlation coefficients (ICCs) reflecting within cluster (care coordinator) and cluster-period correlations were estimated using our preliminary dataset. Following the notation of Hemming et al.,[5] we refer to these ICCs as the between-period ICC (ρ_B) and the within-period ICC (ρ_W), respectively, with the cluster auto-correlation (CAC) defined as ρ_B/ρ_W. First, following recommendations we fit a linear mixed-effect model (LMM) to our binary outcome data[5] with both types of random intercepts and found that the estimated random effect variances were estimated to be zero (corresponding to ICCs of 0 for both). Second, we fit a GLMM and likewise the random effect variances to be equal to zero. Both the LMM and GLMM included a fixed effect for period. However, just because our estimated ICCs were zero does not mean that the true ICCs are zero, so we also considered sensitivity scenarios in which we varied these values. Specifically, we considered a small within-period ICC of ρ_W = 0.01, and we considered values of ρ_B of 0.008 and 0.005 (corresponding to CACs of 0.8 and 0.5, respectively).[4]

With an assumed sample size of 30 care coordinators randomized (15 per allocation sequence), we estimated[5] that we will have ≥80% power to detect an increase in the proportion of patients with SUD treatment engagement of ≥14.4 percentage points in the intervention arm compared to usual care, assuming a baseline outcome rate of 33% (preliminary data); see **Table 6.1** for effect sizes across the different parameter value for the ICCs considered.

Table 6.1 Minimal detectable difference (MDD) in the absolute difference in SUD treatment engagement with >80% power comparing intervention versus usual patients (intention-to-treat [ITT] analysis); **primary scenario bolded**

Outcome %		Difference	ICC	CAC	Power
Usual Care	Intervention		(ρ_W)	(ρ_B/ρ_W)	
**33%**	**47.4%**	**14.4%**	**0**	**0**	**80.4%**
33%	47.7%	14.7%	0.01	0.5	80.4%
33%	47.4%	14.4%	0.01	0.8	80.0%

Because not all patients visiting a coordinator during the intervention period will participate in the care navigation intervention, any increases in SUD treatment engagement will be attenuated by those not participating in care navigation for whom we expect similar treatment rates as seen in usual care (33%); see **Table 6.2** below. Based on our pilot, we expect at least 50% of patients to engage with the navigator. If 50% of patients engage with care navigation, we need to observe a 28.8 percentage point increase in the proportion with treatment engagement (from 33% to 61.8%). Although large, this effect is plausible based on a prior study among primary care patients referred to SUD treatment. In that prior study, a recovery management checkup intervention that included core components of care navigation resulted in 46% of patients being linked to SUD care within 3 months compared to 20% in the control group.[8]

Table 6.2 Minimal detectable difference (MDD) in the absolute difference in the proportion with SUD treatment engagement with >80% power comparing intervention versus usual patients (intention-to-treat [ITT] analysis); and corresponding MDD among patients who engage with the care navigation intervention, across levels of engagement.

ITT	Engaged patients (%) versus usual care			
	50%	60%	70%	80%
14.4%	28.8%	24.0%	20.6%	18.0%

7.0 DESCRIPTIVE ANALYSES

7.1 Analyses of Demographic and Baseline Data

The demographic variables for this study include age, sex, race and ethnicity, community characteristics (e.g., census variables), and type of insurance. The baseline clinical characteristics include diagnoses of medical conditions, mental health disorders, substance use disorders, and receipt of prior SUD treatment. Care coordinator-level variables include aggregated measures of patient-level variables (e.g., proportion female) as well as care coordinator cluster size (e.g., number of eligible patients seen).

Descriptive statistics for baseline and demographic variables will be presented for the randomized care coordinators and for participants assigned to the care coordinators, overall and separately for each allocation sequence, as well as separately for each intervention period. Descriptive statistics will include N, mean, standard deviation, median, 25th and 75th percentiles, minimum and maximum for continuous variables and proportions and percentages for categorical variables. Since randomization is expected to produce balance at baseline across allocation sequences, statistical comparisons of treatment groups with respect to baseline characteristics will not be conducted.

7.1 Crossover

We will examine crossover of patients between care coordinators and between usual care and intervention periods. Specifically, we will report the proportion of eligible patients visiting a care coordinator during one intervention period who also had an eligible visit to a care coordinator during a usual care period.

Because it is possible that some patients visiting care coordinators during the trial eligibility period will have previously seen (the same or different) care coordinator(s) during the pilot phase (i.e., pilot eligibility criteria), we will additionally examine whether any patients eligible for the trial were previously offered the care navigation intervention (during the pilot period).

8.0 OTHER CONSIDERATIONS

8.1 Missing Data

Given that the primary outcome relies on EHR and healthcare claims data on SUD treatment, if there is no evidence of a particular event, we will assume that the event did not occur.

We expect patients who are enrolled in the KPWA health plan at the time of the index visit (and who remain enrolled over the follow-up period) will have complete capture of the primary outcome. In addition, KPWA cares for patients on Medicaid who may also be seen at the mental health access center. For these Medicaid patients, we will have EHR data on treatment but no claims data; however, we will seek to obtain outcome data from Washington State to capture SUD treatment visits billed to Medicaid. If we are not able to obtain Medicaid data, we plan to exclude Medicaid-enrolled patients from primary analyses. Patients not enrolled in KPWA health plan or in Medicaid are not eligible (see Section 3.0), because we do not know when outcome data are missing or not.

If patients disenroll from the health plan during the outcome follow-up period, then we will not have complete capture of the outcome. We plan to examine whether the proportion of patients who disenroll during the outcome follow-up period differs depending on whether the patient visited during the intervention vs usual care period. If we observe that >10% patients disenroll during the outcome follow-up period, we will consider time-to-event analyses (using Cox proportional hazard models), adjusting for baseline factors that are associated with disenrollment or with the outcome.

It is also possible that some of the care coordinators randomized for the trial may leave the health system. We plan to examine whether there are differences in baseline characteristics of providers who drop out versus those continuing in the study, and we will consider adjusting for baseline factors associated with drop out in the regression model.

9.0 SAFETY AND INTERIM ANALYSES

Due to the nature of this study—testing implementation interventions, with all care provided by the health systems and using only secondary data—there are no formal interim analyses of safety performed. Further, all clinical care—and therefore responsibility for the quality of care—in this cluster-randomized, crossover, pragmatic, quality improvement trial is provided by the health systems. Therefore, no formal interim analyses linked to stopping rules will be conducted. Since all care is provided by the health system, not the study, it would not be appropriate to intervene at the patient or care coordinator level for any safety issue.

REFERENCES

1. Initiation and Engagement of Substance Use Disorder Treatment. NCQA. Accessed December 5, 2024. https://www.ncqa.org/hedis/measures/initiation-and-engagement-of-substance-use-disorder-treatment/

2. Hemming K, Taljaard M, Weijer C, Forbes AB. Use of multiple period, cluster randomised, crossover trial designs for comparative effectiveness research. *BMJ*. 2020;371:m3800. doi:10.1136/bmj.m3800

3. Morgan KE, Forbes AB, Keogh RH, Jairath V, Kahan BC. Choosing appropriate analysis methods for cluster randomised cross-over trials with a binary outcome. *Stat Med*. 2017;36(2):318-333. doi:10.1002/sim.7137

4. Arnup SJ, McKenzie JE, Hemming K, Pilcher D, Forbes AB. Understanding the cluster randomised crossover design: a graphical illustration of the components of variation and a sample size tutorial. *Trials*. 2017;18(1):381. doi:10.1186/s13063-017-2113-2

5. Hemming K, Kasza J, Hooper R, Forbes A, Taljaard M. A tutorial on sample size calculation for multiple-period cluster randomized parallel, cross-over and stepped-wedge trials using the Shiny CRT Calculator. *Int J Epidemiol*. 2020;49(3):979-995. doi:10.1093/ije/dyz237

6. Turner RM, White IR, Croudace T. Analysis of cluster randomized cross-over trial data: a comparison of methods. *Stat Med*. 2007;26(2):274-289. doi:10.1002/sim.2537

7. Li F, Forbes AB, Turner EL, Preisser JS. Power and sample size requirements for GEE analyses of cluster randomized crossover trials. *Stat Med*. 2019;38(4):636-649. doi:10.1002/sim.7995

8. Scott CK, Dennis ML, Grella CE, Watson DP, Davis JP, Hart MK. Using recovery management checkups for primary care to improve linkage to alcohol and other drug use treatment: a randomized controlled trial three month findings. *Addict Abingdon Engl*. 2023;118(3):520-532. doi:10.1111/add.16064
