# Supplementary material for: Design of a cluster-randomized, hybrid type 1 effectiveness-implementation trial of a care navigation intervention to increase substance use disorder treatment engagement: study protocol
Source: Addict Sci Clin Pract. 2025 Oct 1;20:78. doi: 10.1186/s13722-025-00605-7 (PMC12486859; doi:10.1186/s13722-025-00605-7)
Supplement: Supplementary file 7 — Supplementary material 7: Conceptual Framework for Enhancing Implementation Success. [file 13722_2025_605_MOESM7_ESM.docx]

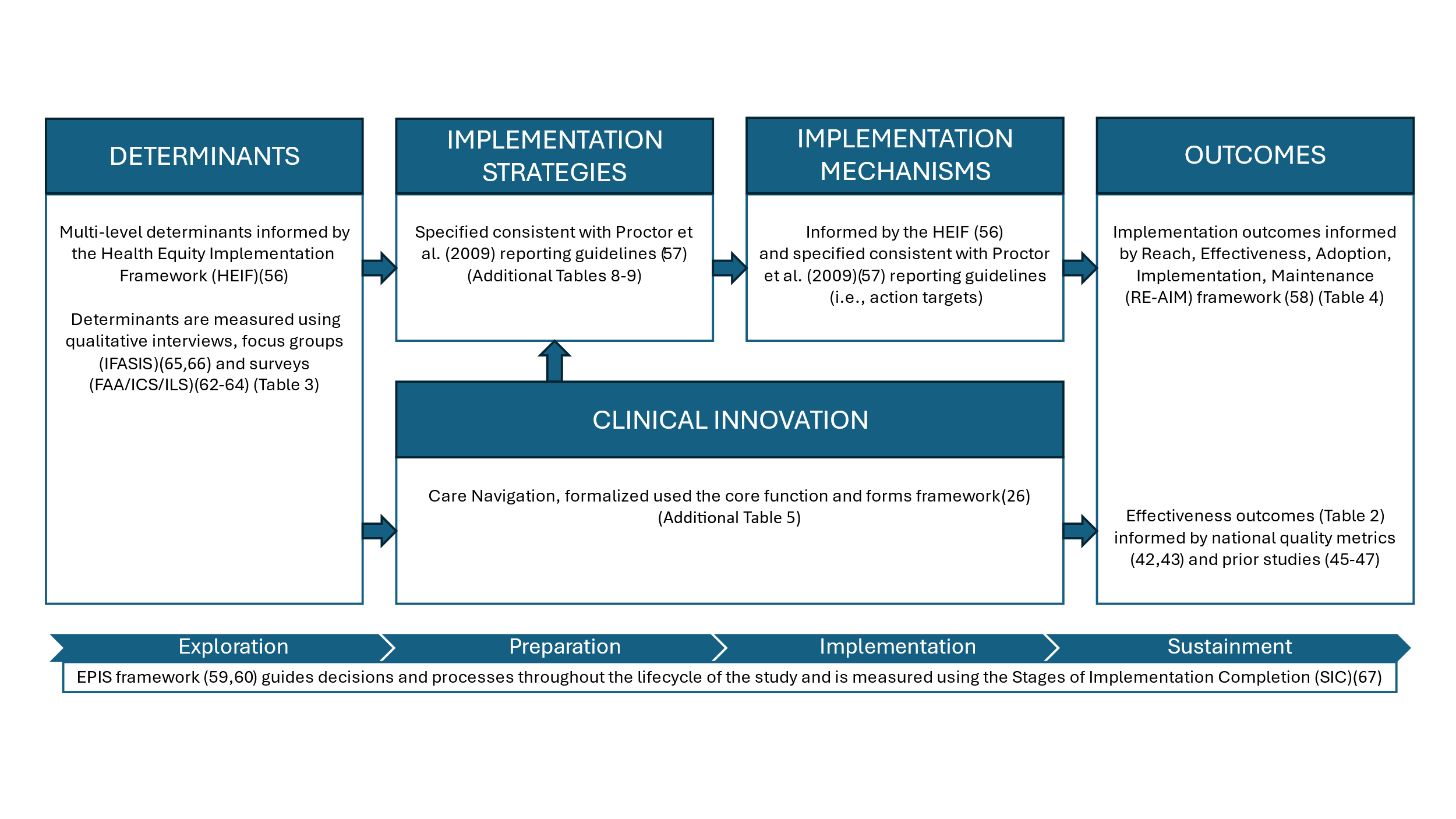


**Additional File 7.** Implementation Research Logic Model adapted from Smith et al. (2022) (55) to visually convey how different conceptual models and frameworks informed the design and implementation of care navigation in the ABC-SUD trial. Models and frameworks are detailed below.

#### Conceptual Frameworks for Enhancing Implementation Success

This design and implementation of this study aligns with the Exploration, Preparation, Implementation, Sustainment (EPIS) Framework (59,60). EPIS identifies factors and processes relevant to each of the four implementation phases across organizational levels and served as the guiding conceptual framework throughout the lifecycle of the study. Formative work to understand contextual determinants is part of the EPIS exploration phase, while care navigation design and trial evaluation are squarely situated in the EPIS preparation and implementation phases, respectively. While sustainment is not the focus of this study, we will seek to understand factors influencing health system decision formally adopt this program. Understanding the challenges and successes experienced during these stages of implementing care navigation in a healthcare system can accelerate the process for future implementers in new settings to adopt, implement, and sustain care navigation. We will use the Stages of Implementation Completion (SIC)(67) to define and measure completion of implementation activities across EPIS phases.

The Health Equity Implementation Framework (HEIF)(56) highlights the potential impact of multilevel factors of disparities in SUD treatment engagement. As part of the EPIS exploration phase, HEIF informed qualitative interviews with patients and providers (see Study Redesign) conducted to better understand contextual determinants at the study site. Specifically, we leveraged two HEIF domains, *culturally relevant factors of patients/clinicians* and *societal context*, to define barriers to treatment initiation that could arise from community or structural inequalities, such as economic hardship, living in rural areas, having lower digital literacy, needing healthcare information in languages other than English, and being a member of racial and/or ethnic groups that have experienced decreased opportunities for successful treatment engagement. In the preparation phase, we designed care navigation protocols that directly act upon these determinants to improve the initiation and engagement of SUD treatment.

The Proctor et al. (2009) framework (57) provides a structure for conceptualizing and specifying implementation strategies and the mechanisms by which they impact implementation outcomes. For each strategy, the actor, action, target, temporality, dose and intended outcome is defined (see Additional Files 8 and 9). As part of the EPIS preparation phase, implementation strategies were selected from prior literature (61) and prioritized based on contextual determinants and implementation mechanisms identified from qualitative interviews during the preparation phase. Specifying implementation strategies using the Proctor et al. (2009) framework enables comparability and replication in future studies and can help articulate plausible causal pathways—how and why a strategy is expected to influence implementation outcomes.

Implementation outcomes are conceptualized using the RE-AIM framework (58). This widely-used framework focuses on five key outcomes relevant to implementation success: Reach (sometimes called penetration), effectiveness, adoption, implementation (which can include fidelity and costs), and maintenance (sometimes called sustainment). While EPIS guides when and where to focus efforts, RE-AIM specifies what to measure to assess impact and guide adaptation. Together, RE-AIM offers outcome domains that help measure progress across EPIS phases.

Conceptual frameworks and rationale informing the clinical intervention design and evaluation are detailed in the manuscript text: For the core functions and forms framework, see *Experimental Intervention*; for HEDIS rationale, see *Outcome Measures*.
