# Supplementary material for: Design of a cluster-randomized, hybrid type 1 effectiveness-implementation trial of a care navigation intervention to increase substance use disorder treatment engagement: study protocol
Source: Addict Sci Clin Pract. 2025 Oct 1;20:78. doi: 10.1186/s13722-025-00605-7 (PMC12486859; doi:10.1186/s13722-025-00605-7)
Supplement: Supplementary file 8 — Supplementary material 8: Specification of care-delivery facing implementation strategies according to Proctor et al. (2011) reporting guidelines. [file 13722_2025_605_MOESM8_ESM.docx]

| **Additional File 8.** Specification of care-delivery facing implementation strategies according to Proctor et al. (2011) reporting guidelines | | | | | | |
| --- | --- | --- | --- | --- | --- | --- |
| **Strategy^1^** | **Actor** | **Action** | **Target(s) of the action** | **Dose and temporality** | **Outcomes affected** | **Justification** |
| Leadership engagement | MHAC leaders and study team | 1. Seek to understand gaps in SUD care and MHAC needs through local consensus discussions 2. Co-design CN by proposing changes and eliciting ongoing feedback 3. Obtain formal commitments from leaders/managers | 1. Engage MHAC leaders to support local implementation    1. Identify partner(s) and obtain buy-in | Monthly 30-minute meeting with MHAC managers and ad hoc meetings (or email) with director | Clinician adoption, implementation  Maintenance | Managers can encourage clinician participation in trial and subsequent referrals for CN. Managers can solve system barriers that arise. Director and other MH&W leaders will determine wither intervention is sustained. |
| Involve patients and care coordinators in intervention and workflow redesign | patients,  pilot care coordinators, MHAC leaders | 1. Assess for readiness and tailor the intervention by interviewing patients and care coordinators about barriers and facilitators to SUD treatment and their perspectives on CN intervention 2. Identify 4 care coordinators as early adopters to learn from their experiences offering CN at MHAC 3. Pilot implementation of CN with these early adopters using small tests of change to specify ideal workflows, problem-solve and adjust activities for improved collection of performance data 4. Conduct post-pilot interviews informed by user-centered design principles with patients who participated in the pilot to obtain and use consumer feedback on CN | 1-2. Ensure feasibility of CN activities to care coordinators  1-3. Tailor intervention and implementation of CN to local context   1. Ensure acceptability and appropriateness of CN activities to patients | Interviews occurred 10/23/2023-1/17/2024 (patients), 10/4/2024-present (care coordinators), and the pilot occurred 7/9/2024-11/27/2024 | Clinician adoption, implementation  CN implementation | Redesign is responsive to changes in the practice ecosystem and external environment, creates new partnerships, and integrates preferences of constituents |
| Develop and implement EHR tools for quality monitoring | Epic programmer with input from MHAC leaders and care coordinators | 1. Integration tools to that automatically prompt care coordinators to offer CN into MHAC assessment templates 2. Provide suggested scripting to care coordinators to increase patient demand for CN 3. Create discrete documentation fields in the EHR to allow for better assessment of patient eligibility | 1-2. Make it easy for care coordinators to offer CN to patients   1. Ensure documentation fidelity in assessment templates | Prepared in advance of trial launch and updated as needed on a regular basis. | Clinician adoption, implementation | Care coordinators are more likely to make referrals to CN if the process is integrated into the systems they already use. |
| Clinician training | care coordinators | 1. Facilitate educational outreach visit by care delivery leaders on assessing and diagnosing SUD 2. Conduct ongoing training on the implementation, including ≥2 educational meetings on making a referral to CN 3. Develop and distribute educational materials (e.g., huddle cards, scripts) that provide details of CN and how care coordinators can offer CN to patients 4. Make training dynamic by setting up a Teams channel for real-time communication with peers and study staff about CN | 1. Increase care coordinator confidence in assessing SUD    1. Increase care coordinator knowledge about CN and the processes for referral 2. Encourage social diffusion of CN among care coordinators | 1. One 1-hour training occurring the week before trial launch 2. Two 30-minute trainings occurring on trial launch and crossover dates (more may be provided as appropriate)   3-4. Prepared in advance of trial launch and updated/monitored as needed on a regular basis | Clinician adoption | Care coordinators are gate keepers to CN and need to know the rationale and processes for referring patients |
| Performance improvement | care coordinators | 1. Protect clinician time at regular team meetings to discuss implementation of CN (i.e., ABC-SUD becomes standing agenda item) 2. Share patient stories that demonstrate benefits of CN 3. Collect and share performance data with care coordinators that summarize referrals to CN and highlight missed opportunities 4. Continue to conduct small tests of change and elicit feedback from care coordinators to refine implementation processes | 1-2. Engage care coordinators to support local implementation   1. Feedback on measurable goals to improve individual performance 2. Problem solve and adjust activities for continuous improvement | Once per month for about 10 minutes as a standing agenda item on bi-monthly team meetings. | Clinician adoption,  implementation  Patient adoption, reach | By having a study presence at team meetings, care coordinators will be reminded that this service exists and will open avenues for bi-directional feedback to further measurable goals |
| Electronic learning collaborative | care coordinators, care navigators | 1. Establish and monitor an electronic discussion forum for care coordinators and care navigators | 1. Facilitate information exchange, communication, and support for care coordinators | Ongoing | Implementation fidelity and competency, patient adoption, reach | Providing opportunities for information flow and ongoing learning empowers care coordinators to better utilize the care navigation intervention |
| **Abbreviations:** ABC-SUD=Addressing Barriers to Care for Substance Use Disorder; CN=care navigation; MHAC=Mental Health Access Center  ^1^ Implementation strategies often used more than one ERIC strategy. These are integrated and elaborated on in the activities domain, when relevant. | | | | | | |
