# Supplementary material for: Design of a cluster-randomized, hybrid type 1 effectiveness-implementation trial of a care navigation intervention to increase substance use disorder treatment engagement: study protocol
Source: Addict Sci Clin Pract. 2025 Oct 1;20:78. doi: 10.1186/s13722-025-00605-7 (PMC12486859; doi:10.1186/s13722-025-00605-7)
Supplement: Supplementary file 9 — Supplementary material 9: Specification of interventionist-delivery facing implementation strategies according to Proctor et al. (2011) reporting guidelines. [file 13722_2025_605_MOESM9_ESM.docx]

| **Additional File 9.** Specification of interventionist-delivery facing implementation strategies according to Proctor et al. (2011) reporting guidelines | | | | | | |
| --- | --- | --- | --- | --- | --- | --- |
| **Strategy** | **Actor** | **Action** | **Target(s) of the action** | **Dose and temporality** | **Outcomes affected** | **Justification** |
| Intervention manuals | care navigators | 1. Protocolize procedures and workflows in a manual of operating procedures | 1. Promote standardization of CN across care navigators | Developed prior to trial launch and iteratively updated as needed | CN implementation | Fidelity monitoring promotes standardization of CN and helps track planful adaptions |
| Population dashboard | study programmer and care navigators | 1. Develop and organize quality monitoring systems by integrating clinical EHR, with study algorithms and databases | 1. Provide care navigators information on newly eligible patients and outreach attempts | Prepared in advance of trial launch and updated as needed on a regular basis. | Patient reach | Care navigators are more likely to conduct timely outreach if the process is integrated into the systems they already use. |
| Fidelity monitoring | care navigators | 1. Self-report the quality of motivational interviewing 2. Record barriers encountered by patients and the interventions used to address them | 1. Align care navigator practices with guiding principles 2. Ensure core components of CN are delivered by care navigators with fidelity | After every CN session with patients | CN implementation | Fidelity monitoring promotes standardization of CN and helps document planful adaptions |
| Clinical supervision | care navigators, PI, and clinical supervisor | 1. Provide ongoing peer supervision 2. Review cases with study team 3. Provide formal supervision with clinical manager | 1. Care navigators support one another to improve CN delivery 2. Study team reviews exemplar cases and discuss barriers and facilitators to CN 3. Care navigators maintain professional standards for licensure | 1. Weekly 30-minute meetings 2. Weekly 30-minute meetings 3. Monthly 1-hour meetings | CN implementation | Different types of supervision support different CN needs to ensure CN is delivered with competence |
| Clinical training | care navigators | 1. Obtain motivational interviewing training from certified instructor 2. Obtain Cultural humility training | 1-3. Ensure CNs are competent in the skills needed to delivery CN | Completed by CNs prior to trial launch and updated as needed for continuing education requirements | CN implementation | CNs are licensed clinicians who must be competent in motivational interviewing, knowledgeable about SUD, and able to demonstrate cultural humility |
| **Abbreviations:** CN=care navigation; PI=principal investigator; SUD=substance use disorder | | | | | | |
